# GC-Biased Evolution Near Human Accelerated Regions

**DOI:** 10.1371/journal.pgen.1000960

**Published:** 2010-05-20

**Authors:** Sol Katzman, Andrew D. Kern, Katherine S. Pollard, Sofie R. Salama, David Haussler

**Affiliations:** 1 Department of Biomolecular Engineering, University of California Santa Cruz, Santa Cruz, California, United States of America; 2 Department of Biological Sciences, Dartmouth College, Hanover, New Hampshire, United States of America; 3 Gladstone Institutes, University of California San Francisco, San Francisco, California, United States of America; 4 Center for Biomolecular Science and Engineering, University of California Santa Cruz, Santa Cruz, California, United States of America; 5 Howard Hughes Medical Institute, University of California Santa Cruz, Santa Cruz, California, United States of America; University of Michigan, United States of America

## Abstract

Regions of the genome that have been the target of positive selection specifically along the human lineage are of special importance in human biology. We used high throughput sequencing combined with methods to enrich human genomic samples for particular targets to obtain the sequence of 22 chromosomal samples at high depth in 40 kb neighborhoods of 49 previously identified 100–400 bp elements that show evidence for human accelerated evolution. In addition to selection, the pattern of nucleotide substitutions in several of these elements suggested an *historical* bias favoring the conversion of weak (A or T) alleles into strong (G or C) alleles. Here we found strong evidence in the derived allele frequency spectra of many of these 40 kb regions for *ongoing* weak-to-strong fixation bias. Comparison of the nucleotide composition at polymorphic loci to the composition at sites of fixed substitutions additionally reveals the signature of historical weak-to-strong fixation bias in a subset of these regions. Most of the regions with evidence for historical bias do not also have signatures of ongoing bias, suggesting that the evolutionary forces generating weak-to-strong bias are not constant over time. To investigate the role of selection in shaping these regions, we analyzed the spatial pattern of polymorphism in our samples. We found no significant evidence for selective sweeps, possibly because the signal of such sweeps has decayed beyond the power of our tests to detect them. Together, these results do not rule out functional roles for the observed changes in these regions—indeed there is good evidence that the first two are functional elements in humans—but they suggest that a fixation process (such as biased gene conversion) that is biased at the nucleotide level, but is otherwise selectively neutral, could be an important evolutionary force at play in them, both historically and at present.

## Author Summary

The search for functional regions in the human genome, beyond the protein-coding portion, often relies on signals of conservation across species. The Human Accelerated Regions (HARs) are strongly conserved elements, ranging in size from 100–400 bp, that show an unexpected number of human-specific changes. This pattern suggests that HARs may be functional elements that have significantly changed during human evolution. To analyze the evolutionary forces that led these changes, we studied 40 kb neighborhoods of the top 49 HARs. We took advantage of recently developed DNA sequencing technology, coupled with methods to isolate genomic DNA for our target regions only, to determine the genotypes in 22 chromosomal samples. This polymorphism data showed no significant evidence for adaptive *selective sweeps* in HAR regions. By contrast, we found strong evidence for a nucleotide bias in the fixation of mutations from A or T to G or C basepairs. Our work reveals that this bias in the HAR neighborhoods is not just an historic phenomenon, but is ongoing in the present day human population. This finding adds credence to the possibility that non-selective forces, such as biased gene conversion, could have contributed to the evolution of several of these regions.

## Introduction

Understanding the forces that have shaped the evolution of the human genome is one of the most exciting problems in modern genomics. Two approaches to this problem are focused on identification and characterization of those genomic regions that have evolved the slowest and fastest along the human lineage [1]–[5]. The slowest evolving regions may contain elements that cannot be disturbed without disrupting essential function. The fastest evolving regions may harbor elements whose function is unique to our species lineage. To eliminate non-functional regions, both of these complementary approaches begin with a search for regions that are conserved throughout mammalian history or longer. The ultra conserved elements [1] maintain this conservation along the human lineage, and have been shown to be under purifying (negative) selection [6], strongly suggesting that they are functionally important to our species, although in ways that are still largely unknown.

By contrast, several groups have searched for positive selection along the human lineage by focusing on those previously slowly evolving regions of the genome that have evolved most quickly along the human lineage [4], [5], [7]. These regions, such as those in the set of *Human Accelerated Regions* (HARs) [7], may include some of the genetic changes that make our species biologically unique. Indeed, biological characterization of the topmost elements on this list of candidates has proven fruitful: HAR1 is part of a novel RNA gene (HAR1F) that is expressed during neocortical development [3]; HAR2 (or HACNS1) is a conserved non-coding sequence that has been shown to function as an enhancer in the developing limb bud with the human-specific sequence enhancing expression in the presumptive anterior wrist and proximal thumb [8].

Since the HARs were identified based on an excess of fixed differences between the human reference genome and sequences that are highly conserved among chimp, mouse and rat, such differences could have arisen at any time within the 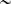
5 million years that have elapsed since our common ancestor with the chimpanzee. As such it is important to recognize that even if such differences resulted from positive selection for advantageous mutations, they may have occurred so long ago that we have little power to find evidence for such selection using only the present day sequences available to us.

Furthermore, as previously noted [7], positive selection might not be the sole explanation for the rapid evolution that is evident in the HARs. Biased gene conversion (BGC) may also hasten the fixation of mutations in a local manner independent of any fitness benefits [9], [10]. BGC arises as a byproduct of recombination between homologous chromosomal regions. In this process DNA double stranded breaks are repaired and the alleles from one chromosome are copied to the other, with a bias for conversion of A or T (weak hydrogen bonding) alleles to G or C (strong hydrogen bonding) alleles [11]–[14]. A neutral locus can thus mimic the rapid evolution of loci under positive selection [9], [10], and furthermore, BGC may in fact drive fixation of deleterious alleles [15], the precise opposite of a positive, adaptive evolutionary effect.

One of the most powerful tools for identifying those regions that have been subjected to directional selection comes from examining the distribution of allele frequencies segregating within a species. For example, analysis of this distribution, known as the *site frequency spectrum* (SFS), allows for the identification of loci that have been involved in selective sweeps in the last few hundred thousand years. Analysis of the SFS has been used to identify targets of natural selection that may be responsible for genetic traits that are uniquely human, such as language [16] or cognition [17].

In the current work we investigate the top 49 HARs that were identified as having a 5% false discovery rate [3]. But rather than restricting our attention to the core elements, which are 100–400bp in length, we consider the polymorphism in a set of 22 chromosomal human samples in a 40kb neighborhood of each of these HAR elements, with an eye to capturing perturbations in the SFS at linked sites, and/or regionally biased patterns of allele fixation. Our samples are drawn from a single population, the Yoruba from Ibidan, Nigeria in order to avoid confounding issues of population admixture as well as to take advantage of a greater degree of variation in this population. We use an adaptation of several techniques previously developed [18]–[21] to enrich genomic DNA from our sample individuals for the target genomic neighborhoods. The enriched DNA is then subject to high throughput sequencing followed by genome-wide mapping of many overlapping sequences to determine genotypes at sites in the target regions, and hence derive the site frequency spectra.

With these spectra in hand, it is possible to test for the hallmarks of BGC. We employed an approach that compares the separate site frequency spectra for the weak-to-strong (i.e. A or T to G or C)(W2S) and strong-to-weak (S2W) mutations to determine if any shift towards high frequency, normally characteristic of a selective sweep, is biased towards one of the two sets of mutations. This signal would indicate an *ongoing* process in the current human population. Similarly, one can compare the proportion of W2S changes among already fixed substitutions on the human or chimp lineage to that among the still segregating sites. A W2S bias in fixed differences relative to polymorphisms would indicate that the regions have historically been subject to a BGC-like biased process.

On the other hand, the spectra may contain evidence of positive selection. Various techniques have emerged in recent years to search for signatures of positive selection using population genetic data [22]–[24], but many are based on the phenomenon of *genetic hitchhiking* [25], [26] in which fixation of beneficial mutations results in a skew in the site frequency spectrum. One such approach [27] is based on the *composite likelihood* of allele frequencies wherein the probability of the observed allele frequency at each polymorphic position is calculated based on its distance from a site under putative positive selection. This probability explicitly takes into account the strength of recombination and selection. A variant of this approach has been implemented [28] in the SweepFinder program that we use herein. It has been previously used [29] in a genome-wide search for sweeps at the scale of 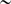
500kb, since that study's data was restricted to loci with common polymorphisms. The power of that approach is probably limited to finding sweeps not much older than 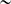
200,000 years but has the attractive property that it is robust to demographic history [29]. Unlike other approaches that have been used [22], [23], [30], [31] it also does not require that the sweep be ongoing or differentially concluded in separate populations. Since we have discovered many novel polymorphisms by resequencing our samples, we use this method to take a more focused look at our 40kb HAR neighborhoods in search of adaptive evolutionary forces.

## Results

We enriched the genomic DNA from 11 individuals among the Yoruba from Ibidan, Nigeria (YRI samples) for 40kb neighborhoods of the top 49 Human Accelerated Regions [7] (HARs) (hereinafter “harseq1-49”), and 13 similar control regions not containing HAR elements (hereinafter “ctlreg50-62”) using a microarray hybridization technique (see Materials and Methods). Using ABI SOLiD [32] high throughput sequencing technology we obtained sufficient coverage in our target regions of the resulting short (35bp and 50bp) sequencing reads to restrict our analysis to genotype calls at positions where we had at least 35-fold coverage for an individual, to ensure accurate genotyping. We determined the frequency of the derived (non-ancestral) allele in our set of 22 chromosomal samples for all the resulting segregating sites (i.e. sites where not all samples have the same allele).

We analyzed these segregating site frequencies in several ways. First, we compared the frequency spectra of two subsets of our segregating sites: those comprising W2S mutations (where the ancestral allele is either A or T, and the derived allele is either G or C) versus the S2W mutations. Next, we compared the ratios of these different classes of mutations to the same ratios for fixed substitutions that have arisen on either the human or chimp lineages since our common ancestor 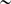
5 million years ago. For both of these analyses we tested the strength of our results across the full scale of our regions, and also performed the tests on the data pooled across our 49 genomic regions. Finally, we examined the pattern of spatial variation of derived allele frequencies in search of evidence for a selective sweep. Results for harseq regions and ctlreg regions were compared to each other. Since the number of control regions (limited by a tradeoff against target regions dictated by the enrichment technology) was small we also compared the results for the target regions to a set of 62 genic regions resequenced in the same 11 YRI individuals by the Seattle SNPs project (see Materials and Methods) [33].

### Weak-to-Strong Mutations Are Being Swept to Higher Derived Allele Frequencies

When the HARs were first described, a strong W2S substitution bias was noted in the human-specific substitutions in these elements [3], [7]. This bias was extremely pronounced in HARs 1,2,3, and 5, but also noticeable as a general trend in the entire set 1–49. This evidence suggested that BGC could have had a historical role in the evolution of the HARs. Here we analyze our list of segregating sites in the 40kb HAR neighborhoods to determine if such bias is still ongoing in the human population. In each region, we separately computed the derived allele frequency spectra for the W2S mutations and the S2W mutations. We then tested for an offset in the spectra between the two categories with a two-sided Mann Whitney U (MWU) test (see Materials and Methods). This test has been shown to have good power to detect fixation bias [34].

We found a significant (p 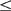 0.05) difference in 11 out of 49 harseq regions (Table 1). In all 11 significant harseq regions, the offset was for the W2S mutations to be segregating at higher derived allele frequencies than S2W. This implies that regardless of the rate of *introduction* of W2S or S2W mutations, it is the W2S mutations that are more likely to reach high frequency and eventually fix in the human population. This is certainly consistent with a mechanism of gene conversion that favors selection of G or C alleles from a heterozygote or some other selective force generally favoring higher GC content. The novel features of this result are that it indicates that this process is ongoing and not confined to the core 100–400bp HAR elements.

**Table 1:** MWU test significant regions.

region	offset	p-value	compare		p-value with mask					recomb		telo
			ctl	sea	500	1kb	5kb	10kb	5kbHi	avg	male	
harseq1*	+1.25	0.03562	0	3	0.03562	0.07069	0.29830	0.72170	0.29830	0.00	0.00	0
harseq18	+3.00	0.02016	0	2	0.08212	0.11110	0.10930	0.19430	0.10930	1.70	1.39	19
harseq20	+1.09	0.01450	0	2	0.00892	0.01128	0.00861	0.01574	0.18860	1.21	1.56	6
harseq21	+2.00	0.00090	0	0	0.00090	0.00090	0.00086	0.00138	0.00510	3.43	4.29	24
harseq25	+1.65	0.00627	0	1	0.00553	0.00826	0.00694	0.01436	0.04611	1.81	0.39	2
harseq27	+1.16	0.01303	0	2	0.01496	0.01496	0.01932	0.12340	0.06593	1.99	1.84	2
harseq32	+0.22	0.02369	0	2	0.03274	0.03140	0.03333	0.01323	0.15330	2.04	1.94	6
harseq34	+1.65	0.00014	0	0	0.00026	0.00030	0.00258	0.00167	0.00258	1.42	0.00	3
harseq35	+1.00	0.04483	0	3	0.04483	0.03461	0.00175	0.01625	0.46510	2.22	1.71	4
harseq43	+1.00	0.01237	0	2	0.01237	0.00721	0.01772	0.05556	0.08562	0.28	0.14	15
harseq46	+1.10	0.02302	0	2	0.01439	0.02300	0.00568	0.00189	0.34770	0.37	0.44	15

Target regions with most significant p-values for Mann-Whitney U test distinguishing the frequency spectra of weak-to-strong (W2S) from strong-to-weak (S2W) mutations. Starred regions are also significant in the MK test (Table 2). offset: the estimated offset of the two spectra, normalized to 22 chromosomal samples, with positive values indicating W2S mutations shifted to higher derived allele frequencies. compare: the number of regions in 13 ctlreg50-62 (ctl) or 62 Seattle SNPs (sea) genic regions with a more significant p-value and positive W2S offset. p-value with mask columns are the p-values derived by omitting all segregating sites at the indicated number of bases from the *center* of the target region. The 5kbHi column gives the highest (least significant) p-value obtained by omitting all segregating sites in a set of overlapping 5kb windows centered at each 2.5kb in the target region. recomb: sex-averaged and male recombination rates from deCODE 1Mb regions. telo: distance of the region from the closer telomere measured in number of karyotype bands, where 0 is the telomeric band.

This ongoing W2S fixation bias distinguishes the harseq regions from ctlreg regions and Seattle SNPs regions. Significant MWU tests were observed at none of ctrlreg50-62 (Supplementary Table 2 in Text S1) and five out of 62 Seattle SNPs regions (Supplementary Table 4 in Text S1), one of which has higher derived allele frequencies in S2W compared to W2S mutations. The distribution of the MWU test statistic in the test regions is also biased towards W2S mutations compared to the 62 Seattle SNPs regions (Supplementary Figure 6 in Text S1). Applying the MWU test to simulations of a neutral coalescent model (see Materials and Methods) showed that the p-values from this test accurately reflect the fraction expected by chance from a neutrally evolving locus (Supplementary Figure 2 in Text S1).

**Figure 1:**
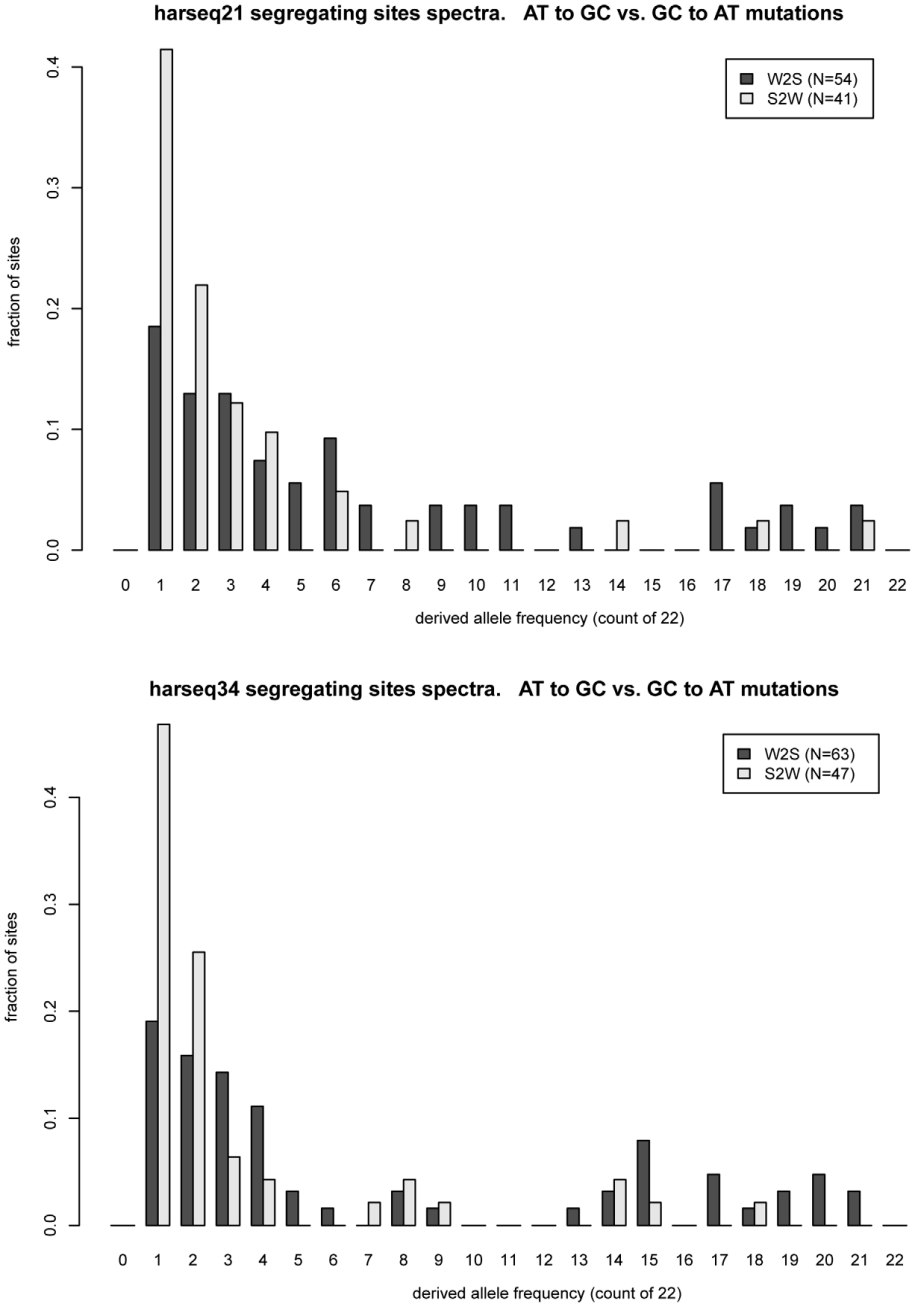
Frequency offset of weak-to-strong versus strong-to-weak mutations. MWU test is strongly significant for harseq21 (upper panel) and harseq34 (lower panel). This reflects the fact that the derived allele frequency spectrum for weak-to-strong mutations (dark bars) is offset towards higher frequencies compared to strong-to-weak mutations (light bars). N: count of segregating sites of the indicated category in the region.

The two harseq regions with the greatest offset were harseq21 and harseq34 (Figure 1). We note that across these two 40kb regions, the *ratio* of W2S to S2W segregating sites is not extreme; it is the W2S shift towards higher frequency in the population that is significant. These ratios are consistent with the smoothed ratios reported in the top several thousand *conserved* candidate HAR elements [7] even though our 40kb regions do not comprise largely conserved regions.

It is natural to theorize that BGC will be a stronger driving force in areas of high recombination and studies have shown there to be a good correlation with the male recombination rate in particular [35]. We examined the recombination rates in the enclosing 1Mb windows as determined by the deCODE project [36]. Harseq21 is an outlier in that it is contained in a genomic region of extremely high recombination rate (male 4.29 cM/Mb, sex-averaged 3.43 cm/Mb, in contrast to genome-wide averages of 0.93 cM/Mb and 1.29 cm/Mb respectively). But this is not true of harseq34 (0 male and 1.42 cm/Mb sex-averaged). For the remainder of the regions with a significant p-value on the MWU test, the rates vary. An additional (not unrelated) factor that has been strongly correlated with biased substitutions is chromosomal position near telomeres [35]. With the exception of harseq1 this is not the case for the regions with significantly shifted W2S spectra (Table 1).

We also performed the MWU test for the shift in W2S sites toward higher frequencies after pooling all the segregating sites in our 49 harseq regions, and found a p-value 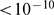. By contrast, the test was not significant (p = 0.26) for the pooled data in our 13 control regions, thereby controlling for possible systematic bias in our sequencing and genotyping techniques. We thus conclude that in many of the neighborhoods of the top 49 HARs there is an ongoing force driving W2S mutations to higher frequency in the human population.

### Weak-to-Strong Mutations Are More Likely to Have Become Fixed Differences

Since the HARs were essentially defined based on fixed differences between the human and chimp reference genomes in otherwise strongly conserved elements [7], we compared such human/chimp fixed differences to the segregating sites in our human samples. Our set of fixed differences was based on high quality base calls from the *reciprocal best* alignments [37] of human and chimp genomes. We further restricted this list to the locations within our regions for which we had the above-mentioned 35-fold coverage for an individual in our sample. Finally, we removed from this initial fixed difference list those positions that we found to be segregating in our samples, or that appeared in the dbSNP129 database [38] (see Materials and Methods). The latter two filters removed 6.7% of the fixed differences at high coverage positions. Since we do not have information on sites that are segregating in the chimp population, we could not remove those, but would expect the number to be similarly small.

We separated the mutations in our segregating site set into the categories: W2S, S2W, and neither. We similarly divided the set of fixed differences, regardless of whether the substitution occurred on the chimp or human lineage. As in reference [35], we performed a variant of the McDonald-Kreitman (MK) test to compare the W2S∶S2W ratios in the sets of mutations and the sets of substitutions (see Materials and Methods).

We found a significant (p 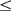 0.05) difference between the substitution patterns of segregating versus fixed sites in 11 of the 49 harseq regions (Table 2). In all but one (harseq39) of these 11, the fixed substitutions had relatively more W2S mutations (compared to S2W) from the ancestral form than did the segregating sites. Four of the 11 fell in the 40kb neighborhoods of the top 11 HARs, and indeed the strongest result (p = 0.00015) was for harseq1. This is not surprising, since the HAR1 element has 18 fixed differences, all W2S [3]. By contrast, none of the ctlreg regions and nine out of 62 Seattle SNPs regions had a significant p-value on this test (Supplementary Tables 2 and 4 in Text S1). All of the nine significant Seattle SNPs regions had a higher W2S∶S2W ratio in fixed differences than in segregating sites. Thus, the harseq regions have a much stronger signal for historical fixation bias than our control regions and a somewhat stronger signal than the genic Seattle SNPs regions. Applying the MK test to simulations of a neutrally evolving primate phylogeny (see Materials and Methods) showed that the p-values from this test accurately reflect the fraction expected by chance from a neutrally evolving locus (Supplementary Figure 3 in Text S1).

**Table 2:** MK test significant regions.

region	p-value	S2W?	compare		p-value with mask					recomb		telo
			ctl	sea	500	1kb	5kb	10kb	5kbHi	avg	male	
harseq1*	0.00015	.	0	0	0.04230	0.03905	0.02225	0.00994	0.02225	0.00	0.00	0
harseq5	0.01073	.	0	5	0.00638	0.01267	0.00623	0.00378	0.14533	1.69	3.39	0
harseq9	0.02186	.	0	8	0.01548	0.01927	0.01493	0.01402	0.15412	2.38	0.33	7
harseq11	0.00079	.	0	1	0.00034	0.00014	0.00122	0.00080	0.00651	2.10	0.00	10
harseq19	0.00394	.	0	3	0.00660	0.02085	0.06709	0.09509	0.07281	0.34	0.05	*1
harseq22	0.04702	.	0	9	0.06066	0.05761	0.10223	0.10821	0.14642	1.11	1.36	3
harseq29	0.04060	.	0	8	0.03907	0.05275	0.13001	0.43571	0.24840	2.52	3.58	0
harseq36	0.02390	.	0	8	0.03300	0.02129	0.09404	0.13464	0.12102	0.34	0.05	*0
harseq38	0.00050	.	0	1	0.00166	0.00145	0.00073	0.00259	0.01245	2.71	2.60	1
harseq39	0.01470	S2W	na	na	0.01957	0.03485	0.04600	0.04711	0.11567	1.99	1.37	2
harseq42	0.00383	.	0	3	0.00509	0.00512	0.02561	0.00465	0.02798	3.12	5.90	0

Target regions with most significant p-values for McDonald-Kreitman-like test distinguishing the fixed substitutions on the chimp or human lineage from the mutations at segregating sites, comparing weak-to-strong and strong-to-weak mutations. Starred regions are also significant in the MWU test (Table 1). S2W indicates that the fixed differences are biased in the direction of strong-to-weak mutations. Other headings as in Table 1. Starred telo values are measured from the chromosome 2 fusion of ancestral telomeres presumed at human chr2:q14.1.

We also recapitulate and expand the finding [7] that this bias towards W2S fixation is associated with telomeres, since 7 of the 11 significant regions under the MK test were found either in the karyotype band containing a telomere or the one immediately adjacent. (This count includes the two cases of harseq19 and harseq36 from chromosome 2 that fall adjacent to the ancestral telomeric fusion event at 2q14.1.) Although many of the 11 regions (including ones near telomeres) have elevated recombination rates, it is also worth noting that 5 of the 11 are in regions with much lower than average male recombination rates (including the two near the chromosome 2 fusion site, and harseq11 on chrX).

One noteworthy *negative* result from the MK test, (and the MWU test as well) is harseq2. It had been noted [7] that the core HAR2 element showed a strong bias towards W2S fixations, and that this extended to a region of 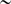
1kb. Here we find no significant signal for either the MK or MWU test in our 40kb neighborhood of that element (Supplementary Table 2 in Text S1). Another noteworthy negative result on these tests is the harseq6 region, which has an extremely elevated rate of mutation as estimated either by nucleotide diversity [39] or by the number of segregating sites [40] (Supplementary Table 1 in Text S1), but apparently no strong bias towards weak-to-strong fixation (Supplementary Table 2 in Text S1).

Although the MK test, like the MWU test, is consistent with the mechanism of BGC favoring fixation of G or C alleles, in fact only one of our regions (harseq1) had significant results in both tests. The complementary evidence from the MWU test (a total of 20 of our 49 test regions are W2S-significant on one test or the other) indicates that the ongoing bias in favor of W2S mutations has also probably led to human specific substitutions in otherwise conserved elements.

### The Scale of Fixation Bias towards Weak-to-Strong Mutations Varies

Because BGC is posited to operate on a scale much smaller than the 40kb of our target sequencing regions, perhaps operating at localized recombination hotspots, and because the (100–400bp) HAR elements at the core of our target regions were suspected of arising in part due to BGC [7], we wanted to test whether the MWU and MK signals depended on these core elements. We therefore performed the same tests after masking out the central 500bp, 1kb, 5kb or 10kb of each region.

We found that signals of both ongoing and historical fixation bias are fairly robust to removing sequences including and flanking the core HAR element. For the MWU test, all but two (harseq1, harseq18) of the 11 regions that were significant at the 5% level for the MWU test were still significant at that level with the central 5kb omitted. Seven of the 11 remained significant even with 10kb omitted (Table 1). For the MK test, the results were slightly more sensitive to masking. Considering the 11 regions with a significant result on that test (including the one favoring S2W fixation), 9(7) of these were still significant with the central 1kb(5kb) masked out (Table 2). We conclude that the evolutionary forces behind these results is not confined to the small HAR elements themselves, but rather that any bias in the substitutions found in the HARs is likely a byproduct of the forces acting at a larger scale.

To test whether there might be other localized elements within the 40kb regions driving these results we performed the tests under a set of fifteen overlapping 5kb masks (centered at a 2.5kb spacing along each region). Among the 11 MWU significant regions, 5 were still significant at 10% under this regime, while 3 completely lost significance (p 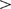 20%) for at least one such mask (Table 1). Of the 11 MK significant regions, 5 were still significant at 10% (including harseq1) under this regime, while 1 completely lost significance (p 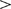 20%) under at least one mask (Table 2). It is worth noting that the harseq1 result reflects the fact that of the 105 segregating sites we found in that 40kb neighborhood, 71 were S2W and only 19 W2S (Supplementary Table 2 in Text S1).

We conclude from this set of tests that the evolutionary forces behind W2S fixation bias are not necessarily highly local. If fixation bias relies on recombination hotspots and BGC, we have to posit that such hotspots extend over a long range of bases, or are somehow temporally and spatially variable (cf. [41]).

### HAR Region Results Are Consistent with Simulations of GC-Biased Evolution

To determine if the results for the MWU and MK tests on the HAR neighborhoods are consistent with a model of GC-biased evolution, we performed simulations under a model of BGC (see Materials and Methods). Of 499 simulations, for the MK test 130 were significant (p 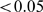) with W2S bias (none significant with S2W bias), and for the MWU test 51 were significant with W2S bias (one significant with S2W bias). The union of the significant W2S simulations on the two tests comprised 169 cases while the intersection comprised 12 cases.

Compared to the simulations, the 49 HAR regions had significantly more than the expected number of MWU W2S cases (p = 0.003 for binomial probability of at least 11/49 cases using the simulation rate of 51/499), but the MK test does not (p = 0.77 binomially comparing 10/49 to 130/499). On the other hand, the small (one case) intersection of MWU and MK tests in the HAR regions is not unexpected based on the simulations. That is, using the fraction 12/499 ( = 0.024) of the MWU and MK intersection in the simulations as the expected rate, the small fraction 1/49 ( = 0.020) in the HAR regions is not statistically significant using either a binomial (p = 0.67) or Poisson (p = 0.67) test. Finally, for neither the simulations (Fisher's Exact test p = 0.74) nor the HAR regions (p = 0.42) is there a significantly greater correlation between the MWU and MK results than expected by chance.

Together, these analyses indicate that the MWU and MK results for the 49 HAR regions are consistent with a model of GC-biased evolution in terms of the overlap between the tests, although the number of MWU cases is enriched compared to the simulation model.

### No Significant Evidence for Selective Sweeps

For each of the studied regions, we used the SFS to calculate two population genetic statistics that can sometimes indicate positive selection: Tajima's D [42], which is based on the *folded* SFS, and Fay and Wu's H [43]. Neither of these statistics exceeded the value of 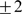 in any region (Supplementary Table 1 in Text S1). We next compared the distributions of these two statistics in the 49 harseq regions and the 13 ctlreg regions, to those for the same population (YRI) in 104 genic regions resequenced by Seattle SNPs (see Materials and Methods). We found the ctlreg regions to be indistinguishable from the Seattle SNPs for these statistics, while the harseq regions were only mildly more negative for Tajima's D (Wilcoxon rank sum p = 0.08) and not significantly different for H (Supplementary Figure 1 in Text S1). These results strongly suggest that the site frequency spectrum in harseq regions is indistinguishable from that found in either our control regions (ctlreg), or in the Seattle SNPs data set. Thus we have no reason to believe that harseqs represent some kind of genomic outlier with respect to recent selective events. Examination of these statistics calculated separately for the W2S and S2W segregating sites (Supplementary Figure 7 in Text S1) shows that the W2S subset in the harseq regions has a significantly more negative value of H than in the Seattle SNPs, which is consistent with the shift to higher derived allele frequencies for this subset noted above using the MWU test.

To test for evidence of a selective sweep, we analyzed the spatial variation of derived allele frequencies at the segregating sites from our 22 chromosomal samples in each of the target 40kb regions using the SweepFinder program. This software determines a composite likelihood ratio (CLR) statistic comparing the hypothesis of a complete selective sweep at the location to the null hypothesis of no sweep using Test 2 from [28]. We tested along a grid of 1000 points in each target region (see Materials and Methods). This test has been shown to be robust to demographic deviations from the standard neutral model in its ability to use an arbitrary background site frequency spectrum [29]. We tested with two such backgrounds: the first from the pooled set of all the data in the 49 harseq regions plus 13 ctlreg regions, the second from the same population (YRI) as our samples but with frequencies taken from the Seattle SNPs resequencing data [33] for a large set (104) of genic regions. It should be noted that using the SFS from our data as the background to define the neutral model should be particularly conservative in that we are testing any given region for deviations from that neutral model. To determine the significance of the maximum CLR values, we performed coalescent simulations of each target region and ran the SweepFinder program on each simulated set of segregating sites (see Materials and Methods). We report as a p-value the fraction of simulations of each target that had a CLR greater than or equal to the actual maximum CLR for that target (Supplementary Table 2 in Text S1)

The harseq regions with the most significant five SweepFinder p-values are listed in Table 3. These are nearly all at the 95% confidence level for either of the two background distributions used, but we note that none are individually significant after a conservative Bonferroni correction, given the 49 harseq regions that were tested. Since these may nevertheless harbor mutations that were selected for in the human lineage, here we briefly note some of their characteristics that can be seen in tracks from the UC Santa Cruz Genome Browser (Supplementary Figure 4 in Text S1).

**Table 3:** SweepFinder hits.

region	p-value		recomb		karyo
	HAR bgrnd	Seasnp bgrnd	avg	male	karyo
harseq9	0.049	0.048	2.383	0.332	chr20.q12
harseq11	0.050	0.043	2.103	0	chrX.p21.1
harseq16	0.052	0.055	0.803	0.679	chr2.q22.3
harseq24	0.113	0.035	0.724	0.247	chr7.p15.2
harseq25	0.026	0.036	1.807	0.387	chr4.q34.3

Target regions with most significant p-values for SweepFinder. HAR bgrnd, Seasnp bgrnd refer to null model background frequency spectrum derived from all target regions or SeattleSNPs respectively (see Materials and Methods). recomb columns are the sex-averaged or male only recombination rates at the target region. karyo is the chromosomal karyotype band where the target region is found.

Unlike the other four SweepFinder hits, which all contain introns or exons of coding genes, harseq25 is in a gene “desert”. The nearest known gene, approximately 1Mb away on chromosome 4, is *ODZ3*, which is a transmembrane signaling protein most highly expressed in brain. Note that harseq25 also has a significant result on the MWU test discussed above.

Evidence for a sweep in the harseq9 region is intriguing because it encompasses the 42-codon long, second exon of the *PTPRT* gene, a phosphatase with possible roles in the central nervous system. However, the human amino acid sequence of this exon matches the other primates chimp, gorilla, and orangutan, except where chimp has an obviously non-ancestral Thr
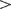
Ala substitution. The human sequence does have a single G
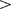
A substitution near the 3′ splice site just upstream of this exon, but it falls in a position between the polypyrimidine (Py) tract and the AG acceptor site, for which the consensus sequence across many splice sites is evenly divided among the 4 nucleotides.

The location of harseq11 on chromosome X places its evidence for a sweep in the first intron of the 2.4Mb long dystrophin gene *DMD*. The evidence for a sweep in harseq16 is offset to one end of its region, about 20kb from an apparent pseudogene comprising a single coding exon with a 270-codon open reading frame (ORF) that is probably derived from the Poly-A binding protein PABPC1.

The harseq24 region encompasses the second through fourth exons of the *SKAP2* gene with the strongest evidence for a sweep about 10kb from the closest exon, but closer to a LINE transposable element that is present also in chimp, orangutan, and rhesus macaque (but lost in gorilla).

Although the above evidence for selective sweeps is not statistically significant, and none of it seems to point directly to a mutation in a core HAR element based on the position of the SweepFinder peak CLR values, it is important to note that while having the advantage of robustness to demography and recombination rate, our tests would not likely have power to detect sweeps that occurred beyond the last 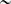
200,000 years [29]. Under an assumption that substitutions in the HAR elements occurred uniformly over the last 5 million years and that most of these substitutions were adaptive, we estimate (see Materials and Methods) that we would be able to detect fewer than 8 with our tests. Therefore this negative result should not be interpreted as ruling out a role for adaptive evolution in the HARs.

## Discussion

In the era of comparative genomics, strong signals of conservation across multiple species serve as signposts that can indicate regions where evolutionary forces may be preserving functional elements that are subject to purifying selection (e.g. [6]). By contrast, signals of positive selection pointing to adaptive changes in one lineage are harder to find, often employing sets of polymorphic sequences from multiple individuals of the same species. We exploited the two recently developed techniques of genomic enrichment and high throughput sequencing to characterize the polymorphism in a single human population across 40kb neighborhoods of the 49 HARs (harseq regions). We investigated the harseq regions because the HARs were defined based on a presumption that the human lineage specific fixed differences therein might have arisen due to adaptive evolutionary forces. On the other hand, it has been emphasized by some that the presumably evolutionarily neutral mechanism of BGC can influence the frequency spectra at polymorphic positions, or cause fixation of alleles in a way that partially mimics the action of adaptive evolution. Indeed, fixation bias was noted in connection with the limited set of human specific alleles for some of the HARs when they were first described [7].

With the extensive novel polymorphism in our samples, we were able to carefully characterize fixation bias — both historical and ongoing — in the harseq regions and to conduct tests for recent selective sweeps across these regions. Our deep resequencing data is noteworthy because it eliminates issues of SNP ascertainment bias that could have skewed previous investigations of polymorphism near HARs. We applied several established population genetic tests, as well as an application of the MWU test, to identify differences in the fixation patterns of W2S and S2W mutations.

Consistent with published reports [7], [9], [35], we find evidence of historical W2S fixation bias in harseq regions. Using a MK test, we compared the proportion of W2S mutations among already fixed substitutions on the human or chimp lineage to that among the still segregating sites in our samples. We found that 11 of our 49 regions show statistically significant evidence of historical bias in allele fixation, with all but one favoring W2S fixation. These results strengthen and expand previous findings by identifying signals for W2S bias in much larger regions flanking the core HAR regions in an ascertainment-free population sample.

This study goes beyond previous approaches by also looking at ongoing W2S fixation bias in the segregating site frequency spectrum. We performed a MWU test using only sites that are still segregating in the human population, separating out W2S from S2W mutations. This second test is designed to detect a phenomenon of bias that is currently driving W2S mutations to higher frequency in the population than S2W mutations. We found statistically significant evidence for this bias (and none in the opposite direction) in the regions flanking 11 of 49 HARs.

For both of our tests, we showed that the core HAR element is generally not the main source of the signal that we detected, since the signal usually remains strong even when we mask out the central 1kb or even 5kb of the region. This is not consistent with BGC due to a recombination hot spot that has remained in the same location for millions of years, because the length scale of the effect of BGC is set by the length of the heteroduplex tract formed during recombination that needs to be repaired, which is thought to be 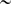
500bp (e.g. [44]–[46]). However, it is consistent with a model in which the location of recombination hotspots drift fairly rapidly over evolutionary time scales, but may be denser in some regions [41], [47]–[50].

It is noteworthy that there was little overlap in the regions identified by these two tests, one for older W2S fixations and the other for present day forces toward fixation, with a total of 20 found in one or the other. Although this is consistent with the hypothesis that the regional focus of BGC, which may be recombination hot spots, drifts significantly on a time scale of many hundreds of thousands or millions of years, we also found from simulations of GC-biased evolution over these time scales that the relatively minimal overlap between the tests is not unexpected.

Another explanation for W2S fixation bias near HARs is selection for increased GC-content or individual fitness-improving GC alleles. To investigate these hypotheses and to attempt to disentangle the possible roles of BGC and positive selection in shaping the HARs, we applied a recently developed powerful method for detecting selective sweeps. Selection was previously investigated in much larger (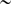
500kb) regions using more sparse polymorphic loci [29]. That study found 101 regions with strong evidence for a selective sweep within 100kb of a known gene. Here, we found only 5 possible candidates for such sweeps among our 49 target regions (and none that were significant after correction for multiple hypothesis testing). Three of these candidates overlap regions with significant evidence of historical (2) or ongoing (1) W2S bias. As we are dealing with a lineage-specific evolutionary period of about 5 million years, and these tests can only see back a few hundred thousand years, it is quite possible that the original signal for selective sweeps in these regions has already decayed beyond our ability to recognize it in human population genetic data. That is, the lack of evidence for recent sweeps does not rule out the possibility that some of the excess substitutions in HARs were fixed by older selection. Similarly, the evidence for GC-biased evolution based on current population genetic data may not fully reflect patterns of polymorphism in the past.

Consistent with the idea that HAR regions may have experienced positive selection too long ago to be detected with population genetic methods, very few positively selected regions in the human lineage have been identified to date, despite the existence of numerous public databases. Selective sweeps that have been identified have typically been the product of very recent events in human history, such as dairy farming affecting the lactase gene [51] or climate differences influencing a salt sensitivity variant [52]. Such environmental or cultural changes result in differences in the genetic makeup of disparate human populations, and such differences can be exploited to find evidence of recent, possibly still ongoing, selective sweeps.

An alternative hypothesis that deserves consideration is that HARs may have an unusually high level of recent substitution due to a recent relaxation in purifying selection along the human lineage (e.g. [53]). Using previously described methods [7], we compared estimates of the rates of substitution in the 49 HAR elements to the neutral rate. We find that the human substitution rate exceeds the expected neutral rate in all 49 HARs, while this is true for the chimp substitution rate in only 10 HARs. Furthermore, in 33 HARs the human substitution rate significantly exceeds the neutral rate (Poisson p-value 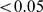) while none of the chimp substitution rates significantly exceed the neutral rate. This evidence argues against the hypothesis that these HAR elements are the product of relaxed selection.

We have focused in our study on 40kb neighborhoods of 49 HAR elements (and 13 similar control regions) because of their intrinsic interest but also because the scope of our study was appropriate to the state of the art of recently emerged enrichment and sequencing technologies. As larger data sets become available we will be able to apply our analysis on a genome-wide scale. Such analysis should give us insights into the properties associated with genomic regions that display this ongoing W2S fixation bias and their potential biological consequences.

Despite the evidence that the unusually high level of recent substitution in the more extreme HAR elements, such as HAR1 and HAR2, could be due to the process of BGC, there is ample evidence that these genomic elements remain functional, and thus the effect of BGC was to mutationally stress but not destroy these elements. HAR1 shows a very strong pattern of compensatory substitutions within its RNA helix structures, indicating a selective force to maintain these helix structures. The W2S substitutions all strengthen the RNA helices of HAR1, and in one case, a substitution appears to extend one of them. Human HAR1 and HAR2 both show evidence of specific function, the former by its highly specific expression pattern during neurodevelopment and the latter by its ability to enhance gene expression during limb development. Whether the human-specific evolutionary changes to these elements reflect a process that was essentially like swimming upstream against an onslaught of non-selective BGC just to keep in place on the fitness landscape, or whether the mutational stress pushed these elements into a configuration that enabled some positive selection for higher fitness in humans, remains to be seen.

## Materials and Methods

### Sample Data Genomic DNA Selection

Genomic DNA for our samples was obtained from the NHGRI Sample Repository for Human Genetic Research distributed by the Coriell Institute for Medical Research [Camden NJ] [54]. All of the 11 samples were chosen from the Yoruba from Ibidan Nigeria (YRI) HapMap population. In particular, the samples were chosen as a subset of the Seattle SNPs P2 panel [33]. The Seattle SNPs PGA-VDR (and Coriell repository numbers were): DY01(NA18502), DY03(NA19223), DY04(NA19201), DY17(NA19143), DY18(NA18517), DY19(NA18856), DY20(NA19239), DY21(NA18871), DY22(NA19209), DY23(NA19152), DY24(NA19210). Sample preparation as described below was similar for all samples, except that prior to processing, the DY01,DY03,DY04 samples were subject to whole genome amplification (WGA) using the Repli-G Kit (Qiagen N.V, The Netherlands) according to manufacturer's specifications. It was found that WGA caused a loss of coverage in some isolated target regions, most notably in the 28kb section of the harseq1 region with coordinates hg18:chr20:61,183,966–61,212,244, except for the core HAR1 element at chr20:61,203,919–61,204,081.

### Target Regions and Nimblegen Enrichment Array Design

The primary target regions consisted of 20kb extensions in both directions from the 49 most statistically significant Human Accelerated Regions (HARs) identified as having a 5% false discovery rate [3]. Additional 40kb control regions were chosen in neighborhoods of 13 of the set of 34,498 vertebrate conserved elements that had extremely *low* LRT scores in the test used to define the HARs. The enrichment arrays were obtained from Nimblegen Systems (Madison WI) who designed the probes on their 385K array based on our specifications of the coordinates of the 62 target genomic regions (Supplementary Table 1 in Text S1). Probes were chosen to tile the target regions from both DNA strands. The design process avoided probes in highly repetitive sequences as described previously [19], [21]. The fraction of bases in the target regions covered by the probes ranged from 98% (harseq12) to 65% (harseq4) with a median of 88%. Details of probed bases are available upon request.

### SOLiD Barcoded Library Preparation

The library preparation of the samples for SOLiD (Applied Biosystems, Foster City, CA) sequencing generally followed the manufacturer's protocols for barcoded SOLiD System 2.0 Fragment Library Preparation (samples DY01,DY03,DY04) and SOLiD System 3.0 Barcoded Fragment Library Preparation (remaining samples), with changes as necessary for enrichment on the Nimblegen arrays as noted in the following: Samples were sheared to approximately 100bp using the Covaris S2 System Program B (Covaris Inc, Woburn, MA). End repair was performed with End-It DNA End-Repair Kit (Epicentre Biotechnologies, Madison, WI) per manufacturer's instructions. Single stranded oligos for the P1 and P2-barcoded SOLiD adaptors were ordered from Invitrogen Corporation and annealed per the SOLiD protocol to form double stranded adaptors, which were ligated to the end-repaired DNA fragments using the Quick Ligation Kit (New England BioLabs, Ipswich, MA) per manufacturer's directions, leaving a nick at the 3′ end of each genomic DNA strand where the 5′ end of the adaptor was not phosphorylated. Samples DY01,DY03,DY04 were size selected to 150
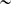
250bp from a 6% polyacrylamide gel and purified via ethanol precipitation. This was followed by nick translation and ligation mediated PCR (LMPCR) amplification (6 cycles) in a combined reaction per the SOLiD protocols using Takara ExTaq polymerase (Takara Bio, Madison, WI). After dividing into 10 aliquots, an additional 10 cycles of ExTaq LMPCR amplification were performed on these samples in preparation for array hybridization. Samples DY17–DY20 were first nick translated without amplification using Pfu polymerase (Stratagene, La Jolla, CA). Samples DY21–DY24 were nick translated and ExTaq LMPCR amplified (6 cycles). Quantitation using a DNA 2100 BioAnalyzer (Agilent Technologies, Waldbronn, Germany) showed that the PCR associated with nick translation *prior* to size selection mainly served to amplify the nick translated adaptors. After the nick translation and any initial LMPCR, all of samples DY17–DY24 were size selected on an E-Gel SizeSelect 2% agarose gel (Invitrogen) per manufacturer's instructions. These size selected samples were then ExTaq LMPCR amplified (6, 9 or 10 cycles) in preparation for array hybridization.

Array hybridization to the Nimblegen arrays was performed on the Nimblegen Hybridization System 4 station under Mix Mode “B” for 64 to 70 hours using the Nimblegen Sequence Capture Kit per manufacturer's instructions. Prior to hybridization, samples DY21–24 were pooled to a total of 5.7
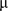
g. All the other samples were hybridized individually, in amounts ranging from 1.9
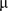
g to 8.0
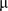
g per sample. For competitive hybridization to probes on the array that might nonselectively bind repetitive DNA, Human Cot-1 DNA (Invitrogen) that had been Covaris sheared to approximately 100bp was added to the hybridization mix in a 5∶1 ratio by weight. Additionally, to block the adaptor ends of the denatured, single stranded DNA fragments from binding to each other, a 10∶1 molar excess of adaptor oligos (P2 with an unused barcode sequence and P1) was also added. At the completion of hybridization, the slide was washed with the Nimblegen Sequence Capture Wash and Elution kit per manufacturer's directions, and the enriched DNA was eluted with 350
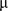
L of 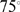
C purified water using an affixed SA200 SecureSeal Hybridization Chamber (Grace Bio-Labs, Bend OR). A secondary elution with an additional 350
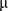
L was also taken. Quantitative real-time PCR (qPCR) was performed on the eluted material to determine the rough fraction of target DNA and to compare the primary and secondary elutions. For this purpose qPCR amplicons within the target were compared to amplicons not in the target regions. It was generally found that the primary elution captured more than 95% of the target DNA (data not shown). By normalizing to pre-enrichment material, and taking into account the fact that the 2.1Mbp target region comprised approximately 0.1% of the entire human genome, it was estimated that more than 35% of the eluted material fell in the target regions (data not shown).

The eluted material was ExTaq LMPCR amplified (samples DY01 for 19 cycles, samples DY03–04 for 15 cycles, pooled samples DY21–24 for 10 cycles, samples DY17–20 for 12 cycles,) in preparation for the emulsion PCR step of SOLiD sequencing that was performed in the UC Santa Cruz Genome Sequencing Center. Samples DY01,DY03,DY04 were processed with the SOLiD Version 2 system, producing 35 bases of sequence information for each read. The remaining samples were processed with the SOLiD Version 3 system, producing 50 bases of sequencing information for each read.

### Sequence Mapping, Filtering, and Genotyping

The 35mer (DY01,DY03,DY04) or 50mer (remaining samples) sequencing reads were mapped to the whole human genome using the *bwa* program [55] which generates mappings and associated quality scores in the *sam* format [56] that can be processed with the *samtools* suite. The *bwa* program is aware of the colorspace nature of the SOLiD sequencing reads, and uses a dynamic programming algorithm to infer the best nucleotide sequence for the read [55]. All reads were also mapped to the DNA of the Epstein-Barr Virus, which was used to transform the Coriell cell lines from which the supplied genomic DNA was extracted. For the female samples, the Y chromosome was excluded from the mapping. For the male samples, the pseudo-autosomal region of the Y chromosome was excluded from the mapping. The set of mappings for each sample was then filtered to the regions covered by the probes on the Nimblegen enrichment array described above. To eliminate spurious pileups caused by overamplification of particular molecules in the library preparation process, the mapped reads were further filtered to select at most 4 reads from each strand at a given genomic starting position. Where there were more than 4, the 4 with the highest total *read* quality (not the best *mapping* quality, which would bias against reads containing non-reference alleles) from the SOLiD instrument were selected. Between 40% and 60% of the reads for a given sample were successfully mapped, and of those reads, between 33% and 48% mapped to bases covered by the probes on the enrichment array (Supplementary Table 3 in Text S1). For samples DY01,DY03,DY04 about 50% of the latter reads were lost in the “maximum 4 per strand” pileup elimination step, while only 11% to 23% were lost in this step in the remaining samples (Supplementary Table 3 in Text S1). This was likely due to the difference in LMPCR cycles used for the different samples as noted above.

To determine the coverage at each position in the target region and the consensus genotypes for each sample, the command “samtools pileup -v” was used with default parameters for its consensus calling model. Possible confounding of the genotypes due to contamination by paralogous sequences was avoided in two ways. First, as noted, only genotypes at positions delimited by the Nimblegen probes were used in the analysis and these probes were designed to avoid repetitive sequences. Second, the bwa mapping algorithm assigns low mapping quality to reads that are not genome-wide unique, and the samtools consensus caller requires high mapping quality. To filter SNPs from among the not homozygous reference genotypes, “samtools.pl varFilter” was run with default parameters, except that the maximum read depth was set to 425, because with up to 4 reads of length 50 on each strand, it was possible to get coverage of 400. This command filters out potential SNPs when more than 2 fall within a 10bp window, on the grounds that there might be an insertion/deletion event rather than separate SNPs, and also filters out reads with RMS mapping quality value less than 25. A similar quality filter was applied to the genotype calls that were homozygous reference. Because of stochastic variation in the composition of reads from the two chromosomes of each diploid individual, low coverage might cause an erroneous homozygous call in a true heterozygote. Therefore a further filter restricted the subsequent analysis to the SNP or homozygous reference calls made for a sample only at positions for which the coverage was 35 or greater. For each target region, the count of the union of such positions across all samples is listed in Supplementary Table 1 in Text S1. As shown in Supplementary Figure 5 in Text S1, the vast majority of the segregating sites that remain after the application of our 35× coverage filter are in Hardy-Weinberg Equilibrium, with only 0.3% having a p-value less than 0.05.

### Derived Allele Frequencies and SweepFinder

For purposes of all subsequent analysis, an ancestral allele at each position in the target regions was determined from the Enredo-Pecan-Ortheus (EPO) pipeline [57], [58] as published on the 1000 Genomes website [59]. This pipeline determines the common ancestor of human and chimp at a locus by considering alignments of the human, chimp, orangutan, and rhesus macaque genomes.

From the sets of filtered genotype calls in the 11 diploid samples as described above all the segregating sites were selected. A set of filters was applied to this list to produce the final set of segregating site derived (i.e. non-ancestral) allele frequencies (DAFs) for all downstream analyses. To avoid skewing the DAFs towards higher frequencies, segregating sites with less than 8 chromosomal samples were eliminated. Also eliminated were any positions with more than 2 alleles among the reference, ancestral, or sample alleles, or where the ancestral allele was not determined by the EPO pipeline. Lowercase values of the EPO ancestral allele, which result from various cases without complete evidence in all species were *not* eliminated.

The *SweepFinder* program [28] was applied to the allele frequencies for the final list of segregating sites to determine the composite likelihood ratio (CLR) of a selective sweep at each one of a grid of 1000 positions across each target region. The model used in this program requires a background derived allele frequency spectrum. Two such backgrounds were used. First, all of the DAFs from all filtered segregating sites in our sample were aggregated and used as input to the command “SweepFinder -f”, which accounts for missing data using a Broyden-Fletcher-Goldfarb-Shanno (BFGS) algorithm. We refer to this as the “harseq” background. A second, presumably more neutral background was obtained from the African-Derived (AD) YRI subset of 24 individuals in the Seattle SNPs P2 panel. The DAFs for all segregating sites in all 104 genes resequenced for this panel by Seattle SNPs [33] were included. As for the harseq background, the “seasnp” background was obtained with the command “SweepFinder -f” applied to these DAFs. The resulting “seasnp” frequency spectrum ran from 1 to 47 and was reduced to a spectrum running from 1 to 21, as needed by SweepFinder with our data, by hypergeometric weighting the relevant components of the input 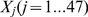 allele frequencies at each target 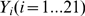 allele frequency.
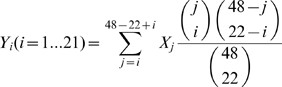
(1)


Dividing by the sum of the 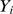 in Eqn 1 produces a valid frequency spectrum that sums to 1. A similar hypergeometric weighting was also required to reduce the spectrum to a range from 1 to 19 for the harseq1,2 and ctlreg60 regions. In the latter regions missing data reduced the maximum number of samples at the segregating sites to 20.

P-values for our SweepFinder results were obtained via coalescent simulation conditional on the observed number of segregating sites in focal region, the observed coverage of this region, and the estimated recombination rate for a given region according to the pedigree data of [36] assuming a human effective population size of 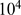. The second point is important here in that our resequencing of both the harseq regions and the control regions was not perfectly complete, but instead was partial owing to an inability to design proper probes for our Nimblegen enrichment procedure (see above) in certain genomic segments. For each region examined we performed 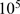 coalescent simulations under the standard neutral model which has been shown to be conservative for the SweepFinder procedure [29].

### Estimate of Number of Detectable Sweeps

From the set of fixed differences between human and chimp in the 49 core HAR elements we use the EPO determined ancestral allele (see above) to count 206 as the total number of human lineage specific substitutions. Since a small number of substitutions are expected to occur by chance even in constrained elements, we used the number of substitutions on the chimp lineage as an estimate of the minimum number of non-adaptive substitutions in each HAR. These total 16 for all 49 HARs. So, we approximate that at most 190 ( = 206-16) substitutions were adaptive in humans. In reality some of the excess nucleotide changes for a given HAR were probably segregating at the same time on the same haplotype. So, 190 is most likely an overestimate of the number of adaptive events in HARs. But suppose there were indeed 190 separate adaptive substitutions and that these occurred uniformly over the last 5 million years. Further assume that any sweep from the last 200,000 years could be detected by SweepFinder. Then, 4% of the 190 adaptive substitutions (i.e., 7.6 sweeps) should be in the detectable time frame. Since the number 190 and the percentage 4% are both upper bounds, we conclude that at most 8 and probably much fewer than 8 sweeps would be detectable by our SweepFinder analysis even if all 49 HARs were shaped by adaptive evolution. Our finding of no significant sweeps after Bonferroni correction and 5 significant before correction is therefore consistent with expectations.

### MWU and MK Tests on Harseq and Ctlreg Regions

To determine if mutations from an ancestral weak (A or T) basepair to a strong (G or C) basepair (W2S mutations) are more likely to spread in the population represented by our samples, we compared W2S segregating sites to S2W segregating sites for each target region and for the aggregate set of segregating sites. We performed a Mann-Whitney U (MWU) test for a difference between the W2S and S2W derived allele frequency spectra. The test was performed in the R language with the command “wilcox.test (paired = FALSE, alternative = two.sided)”. The resulting “location” parameter was normalized to 22 samples and is positive if W2S mutations are segregating at higher frequencies than S2W. The resulting p-values are in Supplementary Table 2 in Text S1.

To determine if relatively more W2S mutations fixed along the human or chimp lineages than are segregating in the human population represented by our samples, we first determined the high mapping quality chimp reference bases that differ from the human reference using reciprocal best alignments of the chimp and human genomes [37]. This set was then restricted to the positions in our target regions for which we had a genotype call for at least one sample with read depth of coverage of 35 or greater as discussed above. From this set of fixed differences we removed any for which the EPO ancestral allele was not determined as discussed above, or for which we had a segregating site, or which appeared in dbSNP release 129 [38]. The remaining fixed differences as well as the segregating sites were divided into W2S or S2W (or other). A McDonald Kreitman-like (MK) test on the resulting 2×2 contingency table was performed in the R language with the command “fisher.test (alternative = two.sided)”. The resulting p-values are in Supplementary Table 2 in Text S1. For the significant cases it was easy to determine from the data in the contingency table if the fixed differences favored S2W mutations relatively more than the segregating sites (column “S2W” in Table 2).

For the target regions with significant p-values on either the MWU or MK tests, we tested whether the significance was due to a restricted locus within the region, by removing all segregating sites and fixed differences under a mask of a given size at a given position within the region and rerunning the test with the remaining data (Table 1 and Table 2).

### MWU and MK Tests on Seattle SNPs Regions and Neutral Simulations

We downloaded the data for the 104 genes resequenced with the Seattle SNPs P2 panel. The genotypes for our 11 YRI samples (which are a subset of the P2 panel) and the coordinates for the genotyped segregating sites were obtained from the global “prettybase” file mapped to UCSC hg18 coordinates. The total set of positions genotyped was obtained from the individual gene “genbank” files by excluding the features defined as “Region not scanned for variation” and aligning the remaining regions to the hg18 coordinates of the full extent of the genic region sequenced specified in the associated “ucscDataFile”. Given these coordinates and the genotypes at the segregating sites, the same techniques as described above for our resequencing data was applied to derive ancestral alleles and human/chimp fixed differences, and to perform the MWU and MK tests.

Our data was derived from (probed) regions of a relatively tight size distribution (Supplementary Table 1 in Text S1). By contrast, the sizes of Seattle SNPs variation-mapped genic regions varied widely. Some were rather small and contained few segregating sites. Therefore, we included only genic regions with a minimum of 10kb variation-mapped and a minimum of 40 segregating sites. Additionally, a small number of the genic regions were excluded because of data missing from the “prettybase” file or because there were no associated high quality reciprocal best human chimp differences as described above, possibly because of paralogous genes in one or the other lineage. The remaining set of results for the MWU and MK tests on 62 genic regions (Supplementary Table 4 in Text S1) were used for comparison to our 49 harseq regions.

To determine if the p-values for the MWU and MK tests were accurate, we also conducted the tests on sets of simulated data under a neutral model. For the MWU test, we performed coalescent simulations using Hudson's *ms* program [60]. For each simulation we generated 22 samples at 85 segregating sites (the average number of W2S plus S2W segregating sites in the 49 harseq target regions) and then randomly assigned the sites as either W2S or S2W in Bernoulli trials using the W2S∶S2W ratio from the 49 harseq regions of 2057∶2114. After calculating the MWU test p-value for each simulation, the fraction of simulations with p-value less than a given value was computed, as well as the subset of that fraction in which the W2S spectrum was offset towards higher derived allele frequencies (Supplementary Figure 2 in Text S1).

For the MK test, for each simulation we separately derived a set of human-chimp fixed differences and a set of segregating sites. The fixed differences were derived using the *phyloBoot* program from the PHAST package [61]. We used a phylogenetic model and substitution rate matrix derived from 4-fold degenerate amino-acid coding synonymous sites across the genome as an unbiased neutral model. The equilibrium GC-content of this model was adjusted to reflect the genome-wide average GC-content. From the primate sequences so generated, we extracted positions containing a human-chimp difference that could also be unambiguously assigned an ancestral allele based on the macaque allele at that position. Each simulation used 335 such sites, (the average number of fixed differences in the 49 harseq target regions) which were divided based on whether they were W2S or S2W (or other). The segregating sites for each simulation were derived from 101 (the average total number of segregating sites in the 49 harseq target regions) Bernoulli trials, randomly dividing them as W2S or S2W (or other) according the corresponding ratios in the fixed differences from all of the *phyloBoot* simulations. After calculating the MK test p-value for each simulation, the fraction of simulations with p-value less than a given value was computed, as well as the subset of that fraction for which the ratio of W2S∶S2W was higher for the simulated fixed differences than for the simulated segregating sites (Supplementary Figure 3 in Text S1).

### Simulations of GC-Biased Evolution

Simulations of GC-biased evolution due to BGC were generated using a forward time Wright-Fisher model of a population. Simulations were run at a population size of 10,000, which is approximately comparable to the long term human effective population size. Details of the simulation method can be found in [62] and references therein. Briefly, we model BGC as a selection process in which each W2S (S2W) mutation adds (subtracts) some normal deviate fitness value to the haplotype on which it is found. This model is approximately equal to the normal shift model [63] if we were only to consider the W2S subset of mutations. Simulations were run for 40 * N generations as a burnin period to reach stationarity, at which point we modeled a vicariance event representing the human chimp divergence. After the population split we ran the two populations for 6.5 units of 4N generations, to approximate the divergence time between humans and chimps. We assume the strength of BGC acting was 4NB = 1.3 as recently estimated from human data [10], [64]. We also assumed a ratio of 4NB∶4Nu of 1. The MWU and MK tests were performed as above using a single sample from the chimp lineage and 50 samples from the human lineage. Association between MWU and MK tests on simulated (and HAR region) data was assessed using Fisher's Exact Test on the 2×2 contingency table defined by the counts of significant or not significant tests: {MWU, notMWU}×{MK, notMK}. The numbers of significant tests by either or both MWU and MK were compared between the simulated and HAR region data using binomial and Poisson tests.

## Supporting Information

Text S1 Supplementary tables and figures.
(0.34 MB PDF)
