# Supplementary material for: GC-Biased Evolution Near Human Accelerated Regions
Source: PLoS Genet. 2010 May 20;6(5):e1000960. doi: 10.1371/journal.pgen.1000960 (PMC2873926; doi:10.1371/journal.pgen.1000960)
Supplement: Text S1 — Supplementary tables and figures. (0.34 MB PDF) [file pgen.1000960.s001.pdf]

Text S1: Supplementary Online Material for: GC-Biased Evolution Near Human Accelerated Regions

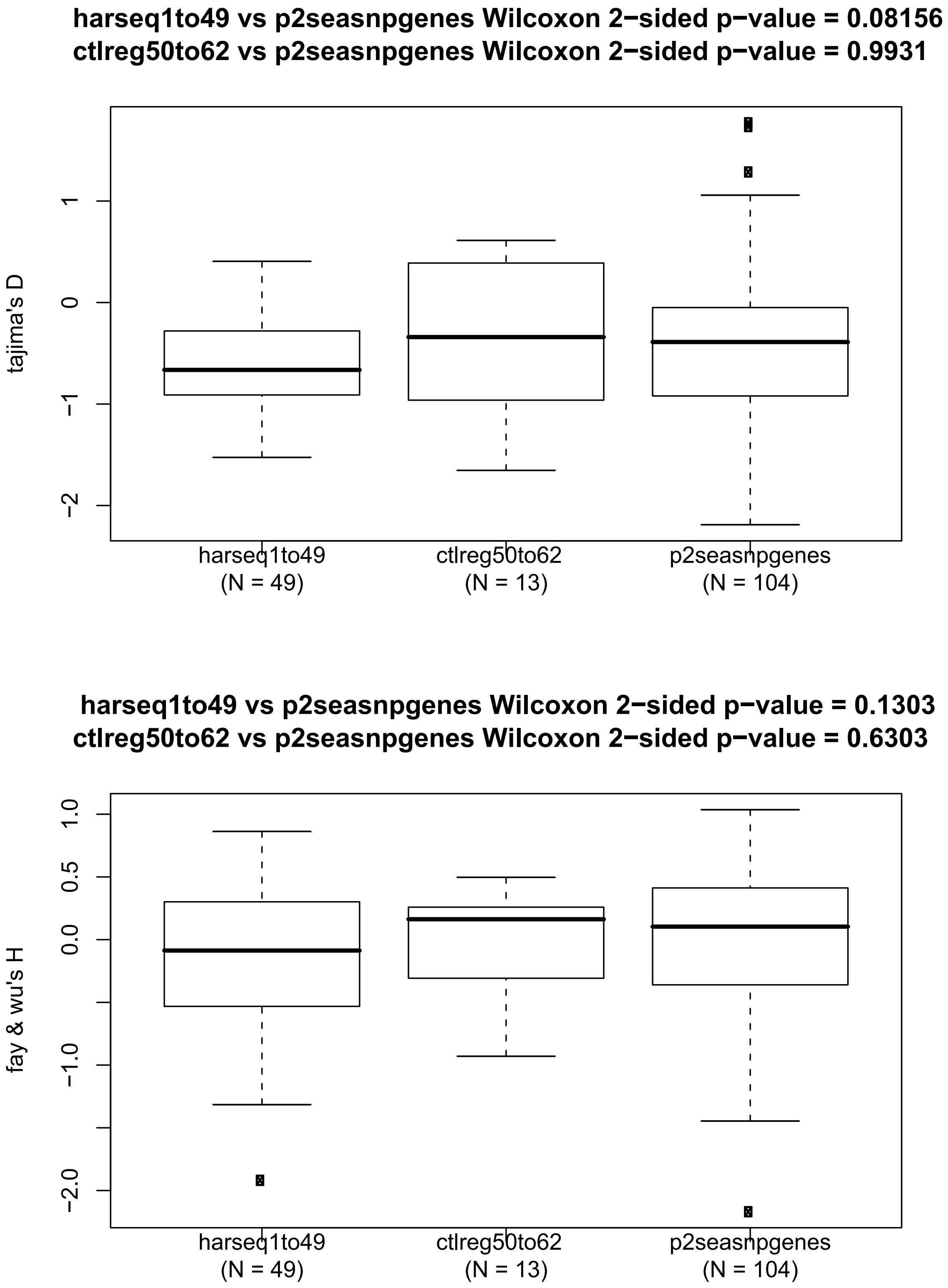

Supplementary Figure 1. Comparison of Population Genetic Statistics in harseq regions and Seattle SNPs genes. Boxplots show the distribution of Tajima's D, Fay and Wu's H in our test regions (harseq1-49), our control regions (ctlreg50-62) and YRI samples sequenced in 104 Seattle SNPs genes.

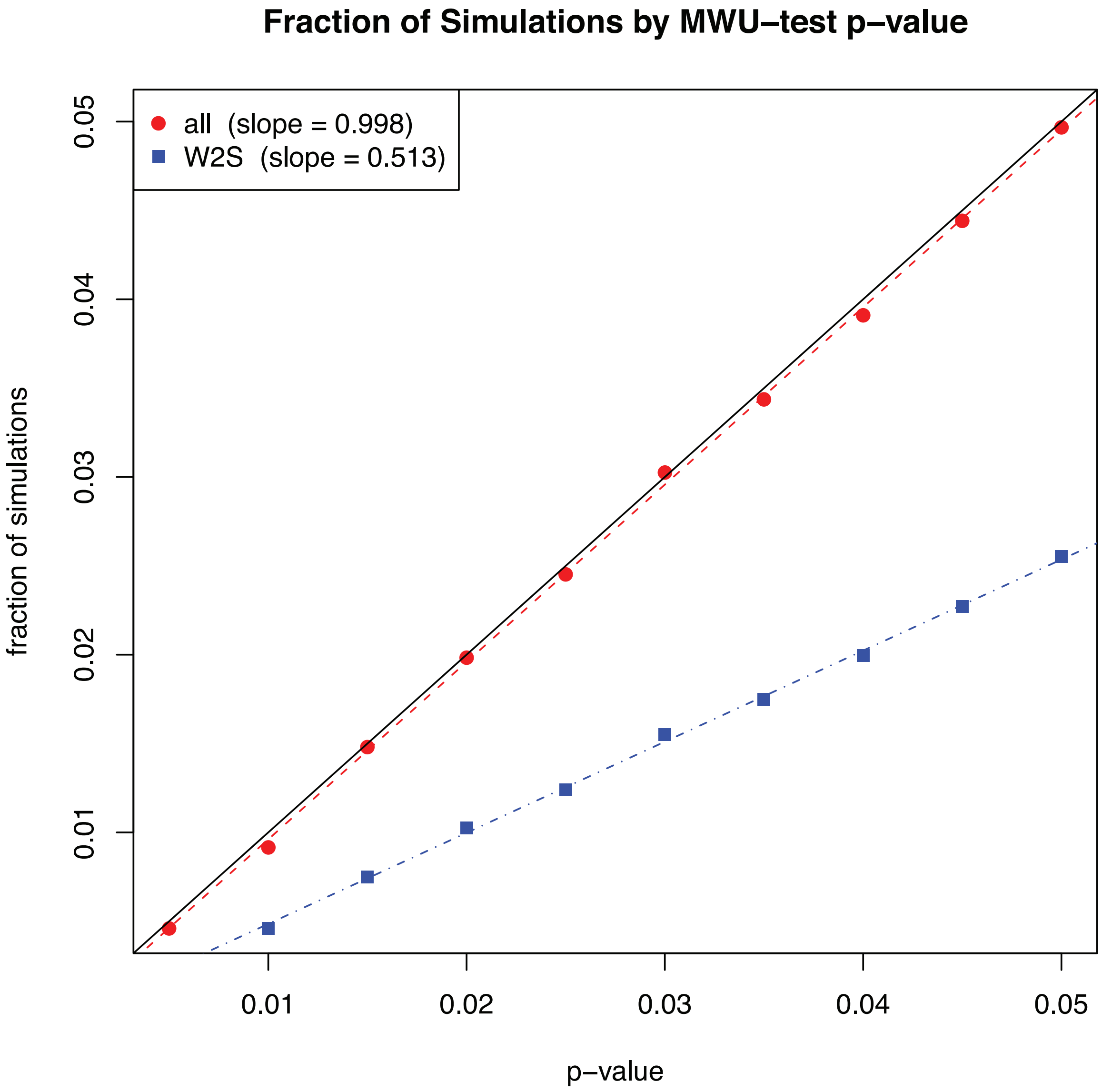

Supplementary Figure 2. Accuracy of MWU Test p-values. For a given p-value the fraction of neutral model simulations (see Methods) having that p-value or better on the MWU test is shown. Red circles: all simulations with a better p-value. Blue squares: the simulations with a better p-value for which the weak-to-strong (W2S) spectrum is offset towards higher frequency. Regression lines are shown with the slope indicated in the legend. For a given p-value the W2S-biased simulations comprise about half of all simulations.

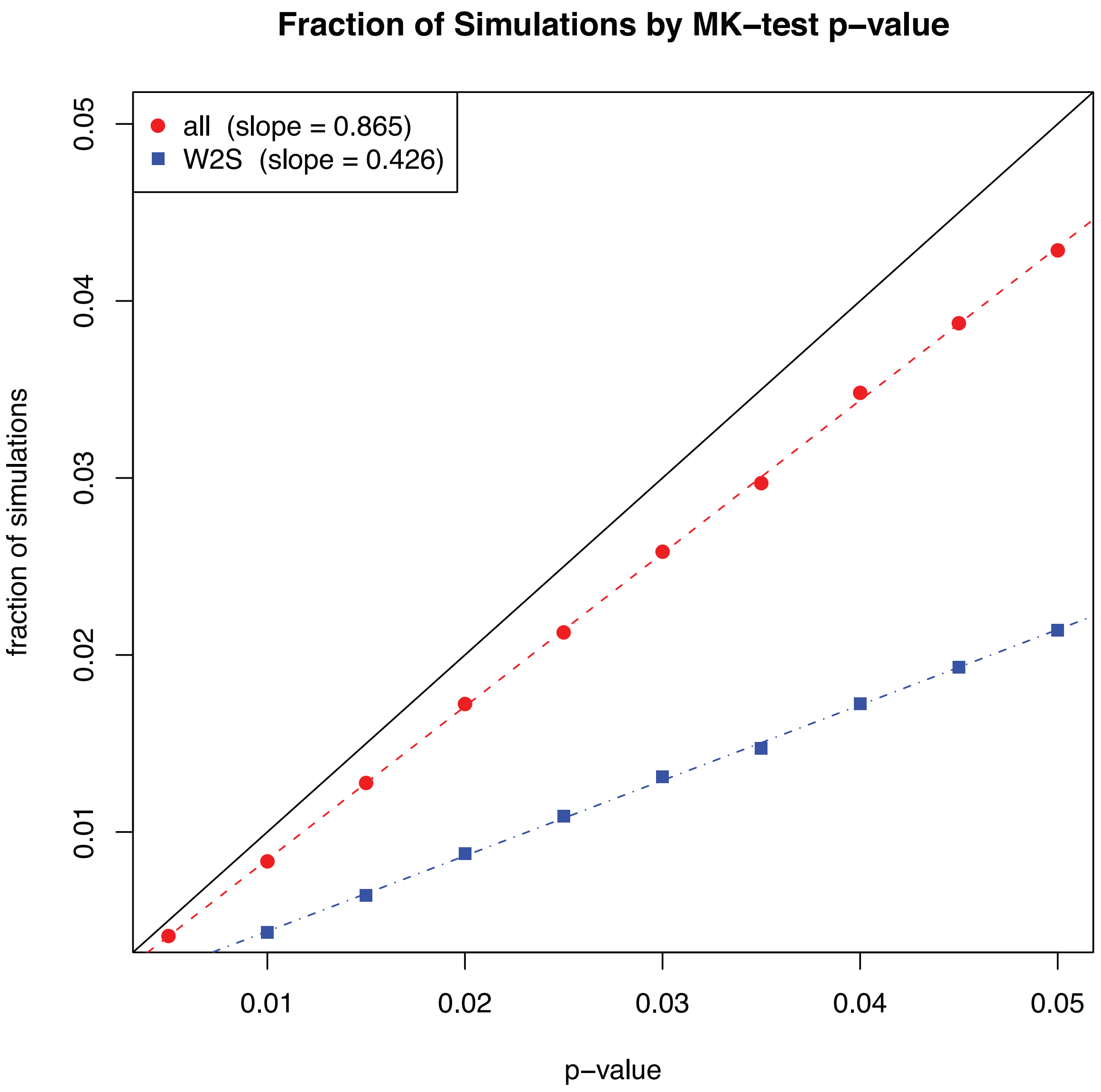

Supplementary Figure 3. Accuracy of MK Test p-values. For a given p-value the fraction of neutral model simulations (see Methods) having that p-value or better on the MK test is shown. Red circles: all simulations with a better p-value. Blue squares: the simulations with a better p-value for which the weak-to-strong (W2S) fraction is relatively higher in fixed differences than segregating sites. Regression lines are shown with the slope indicated in the legend. The p-values are conservative in that they overestimate the fraction of simulations at a given significance level. For a given p-value the W2S-biased simulations comprise about half of all simulations.

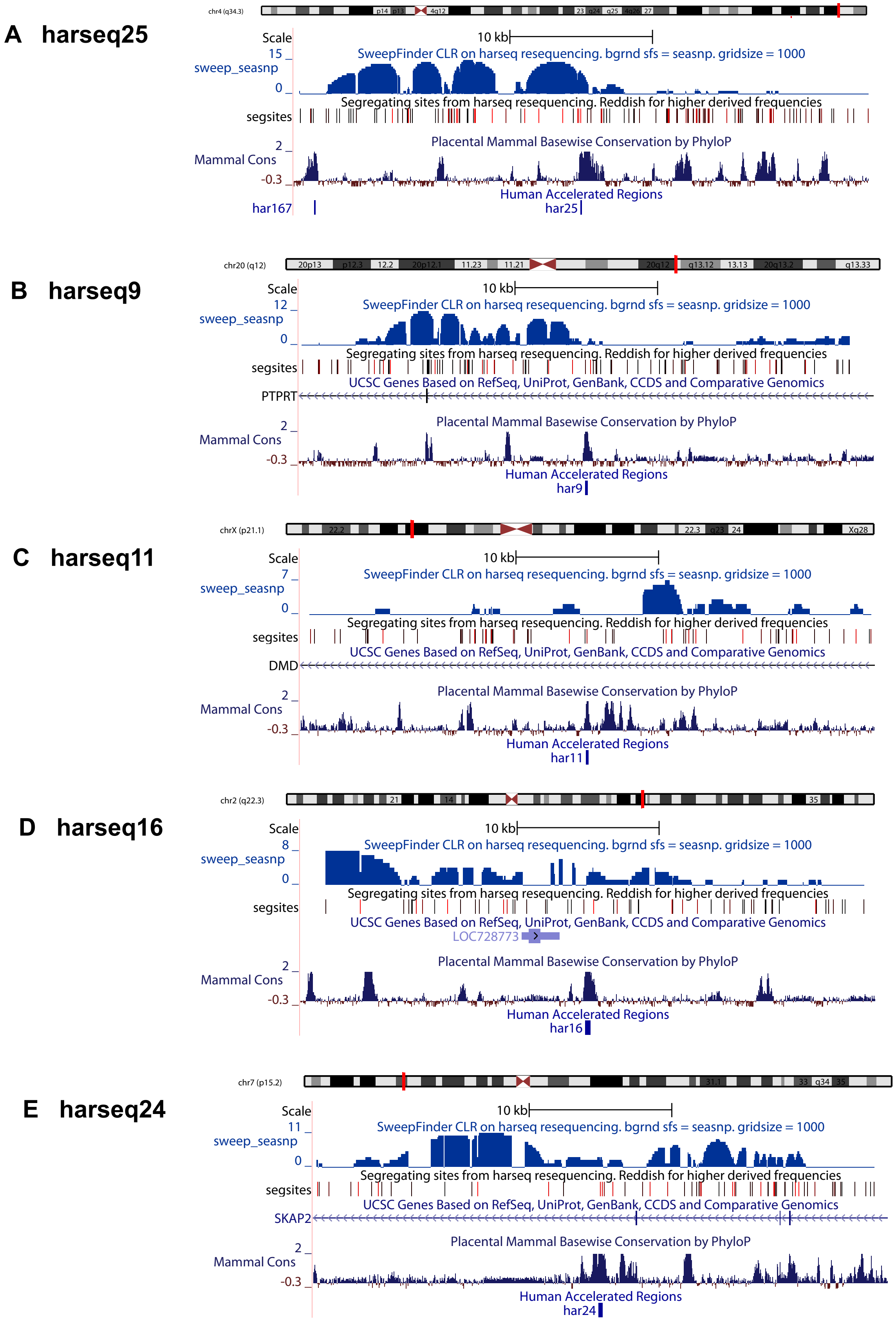

Supplementary Figure 4. SweepFinder Composite Likelihood Ratio (CLR) in genomic context. For the indicated harseq regions, the SweepFinder results are shown along with the segregating sites from our sequencing – more reddish for those with higher derived allele frequencies. Other tracks show introns and exons of known genes and evolutionary conservation.

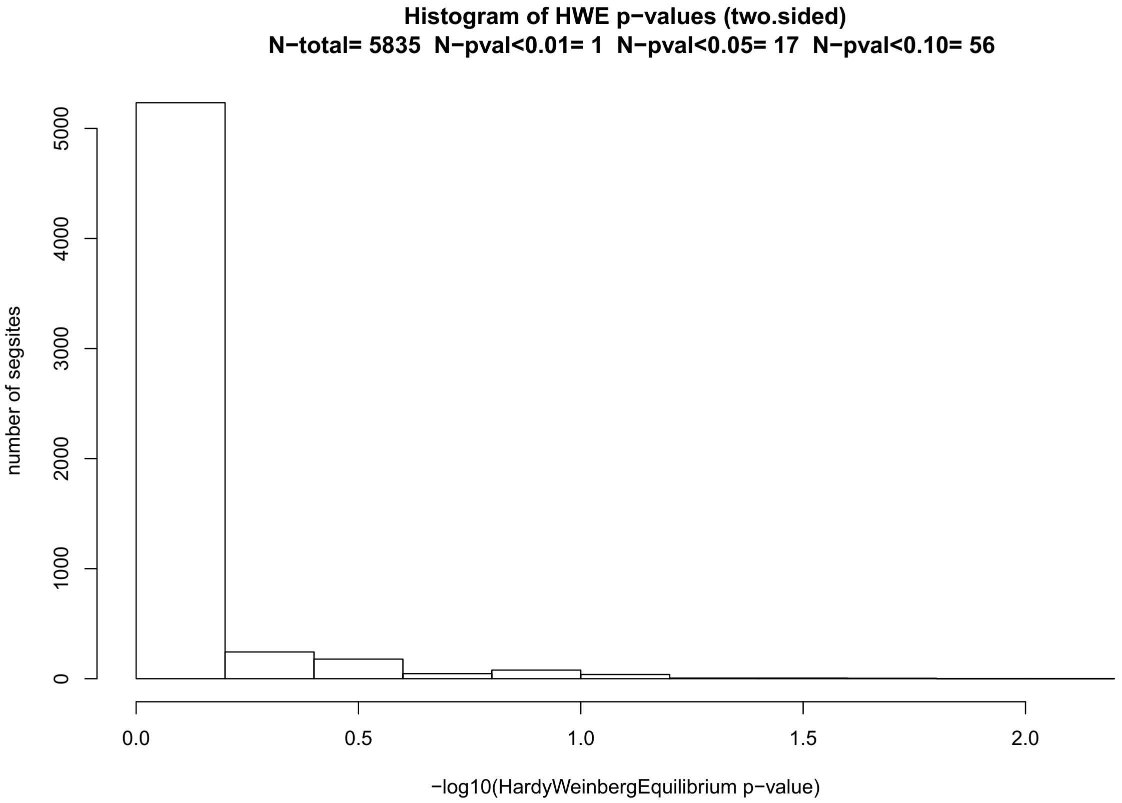

Supplementary Figure 5. Histogram of Hardy-Weinberg Equilibrium Test. Histogram of the p-values obtained in a 2-sided test for Hardy-Weinberg equilibrium across all of the SNPs used in the analysis of our test (harseq) and control (ctlreg) regions.

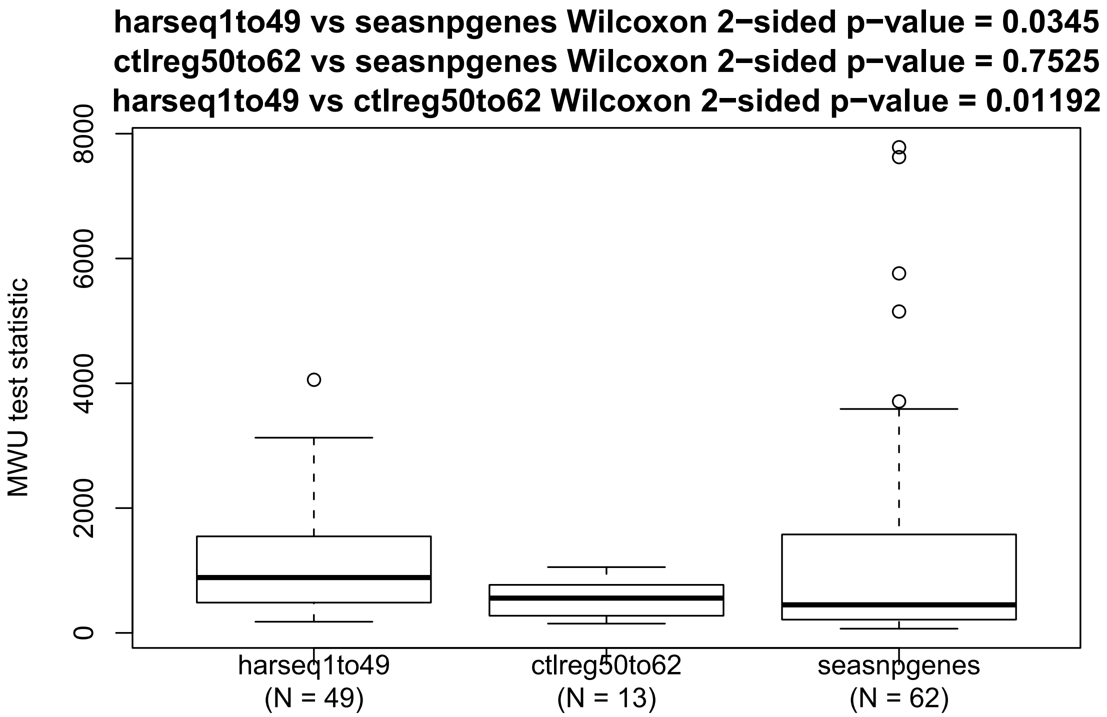

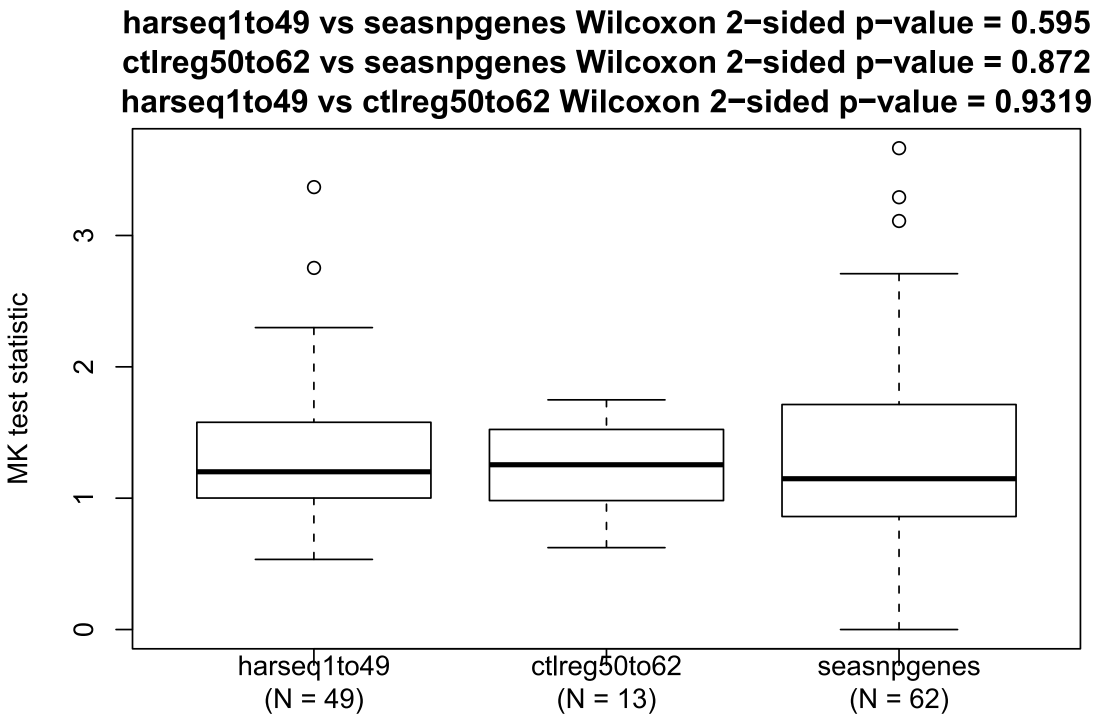

Supplementary Figure 6. Comparison of MWU and MK Test Statistics in harseq regions and Seattle SNPs genes. Boxplots show the distribution of test statistics for MWU and MK tests in our test regions (harseq1-49), our control regions (ctlreg50-62) and for the same samples sequenced in comparable regions for 62 Seattle SNPs genes.

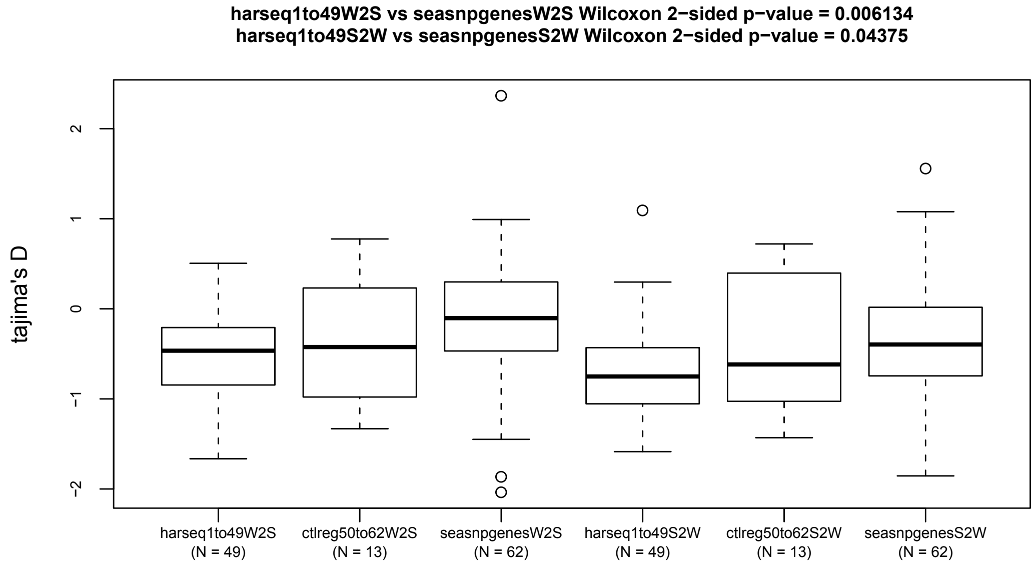

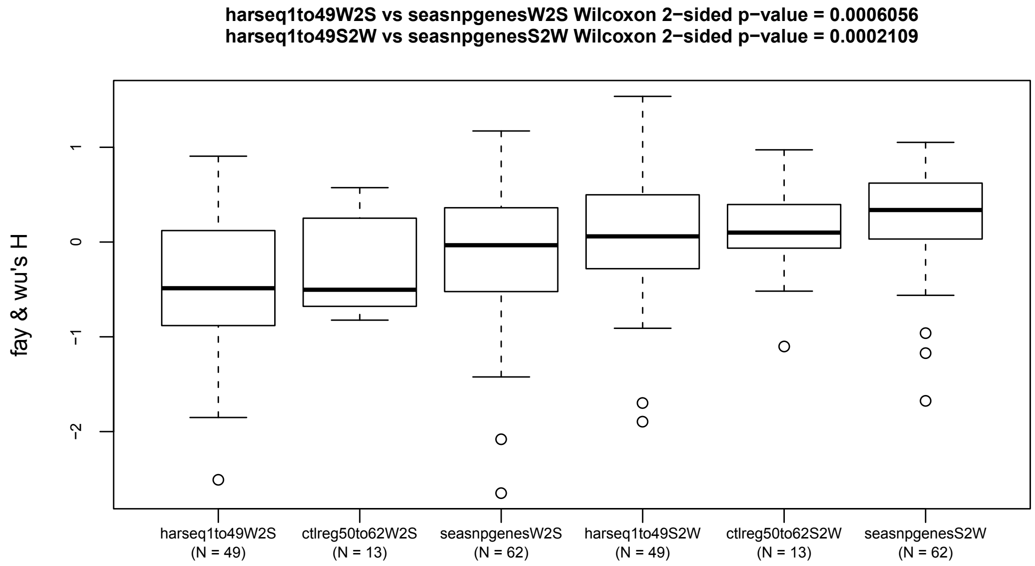

Supplementary Figure 7. Comparison of Population Genetic Statistics for W2S and S2W SNPs. Boxplots show the distribution of Tajima's D, Fay and Wu's H separately computed for weak-to-strong and strong-to-weak SNPs in our test regions (harseq1-49), our control regions (ctlreg50-62) and for the same samples sequenced in comparable regions for 62 Seattle SNPs genes.

Supplementary Table 1. Experiment Region Statistics. Characteristics of the target (harseq1-49) and control (ctlreg50-62) regions in this study. hg18 coordinates: chromosome coordinates in the hg18 human assembly. bpExp: the number of basepairs used in the experimental analysis. This number starts with the count of bases in the region that were included in probes in the Nimblegen array, but only includes bases for which sequencing coverage of at least 35X was reached for at least one sample, after removing pileups of more than 4 reads at the same starting point. See Methods. AncGC: the GC percentage of the *ancestral* bases in bpExp. θ_π: estimate of the neutral mutation parameter ($= 4N_e u$ x10^4) via nucleotide diversity. θ_W: estimate of the neutral mutation parameter via the number of segregating sites. tajD: measure of skew in the folded site frequency spectrum. faywuH: measure of skew in the unfolded site frequency spectrum.

region	hg18 coordinates	bpExp	AncGC	θ_W	θ_π	tajD	faywuH
harseq1	chr20:61183965-61224071	31733	56.2%	10.54047	9.43912	-0.4731	+0.8623
harseq2	chr2:236418752-236458871	34070	46.1%	7.17974	6.71803	-0.2775	+0.0301
harseq3	chr7:2152512-2192618	30022	50.3%	10.72075	8.28494	-0.9819	-0.0811
harseq4	chr16:71666981-71707100	23755	44.2%	9.78895	8.45618	-0.5766	-0.0275
harseq5	chr12:824470-864816	31234	36.1%	7.01946	5.08712	-1.1693	-0.0869
harseq6	chr16:76896721-76937050	31841	41.6%	20.62931	17.40305	-0.6637	-0.2327
harseq7	chr12:59182864-59222966	28472	35.2%	5.60549	5.53491	-0.0521	+0.3392
harseq8	chr7:39171028-39211261	33902	40.1%	7.35471	5.87659	-0.8352	-0.8764
harseq9	chr20:40938987-40979210	31760	43.4%	8.82321	6.30251	-1.1789	-0.5897
harseq10	chr3:60880833-60920936	29045	37.5%	10.16143	11.14925	+0.4055	-0.6030
harseq11	chrX:33034740-33074938	27152	35.6%	7.34905	5.47192	-1.1524	-1.0930
harseq12	chr5:158115485-158155617	37749	38.2%	8.21087	5.50315	-1.3589	-0.3801
harseq13	chr2:177432702-177472931	31665	37.3%	8.46993	7.78707	-0.3345	-0.2526
harseq14	chr11:130450639-130490857	33854	42.6%	14.47888	13.51894	-0.2799	-0.2101
harseq15	chr13:71992984-72033309	32550	38.4%	8.29575	8.41519	+0.0600	+0.1106
harseq16	chr2:147045929-147086345	31192	33.7%	5.67203	3.61549	-1.5250	-0.7265
harseq17	chr6:51381872-51422020	27947	37.4%	7.03796	6.24764	-0.4728	+0.4385
harseq18	chr15:34158826-34199079	30108	36.2%	8.77081	9.06415	+0.1431	+0.3857
harseq19	chr2:118764022-118804131	31255	41.9%	6.93958	6.48426	-0.2705	+0.5394
harseq20	chr4:23464851-23505087	36513	39.4%	9.34632	8.97408	-0.1666	+0.5273
harseq21	chr14:32576700-32616801	35296	37.4%	8.29671	7.25638	-0.5198	-0.0413
harseq22	chr10:10494271-10534383	31680	37.2%	9.99841	9.10019	-0.3763	-0.3379
harseq23	chr4:155948558-155988676	29859	33.8%	9.61144	7.61093	-0.9105	-0.1429
harseq24	chr7:26827593-26867857	28346	34.7%	6.82651	5.33815	-0.9150	-1.9194
harseq25	chr4:182488914-182529055	34372	36.2%	10.52848	8.54024	-0.8011	-1.3152
harseq26	chr12:93433099-93473213	26935	43.7%	8.83290	9.25434	+0.2001	-0.4623
harseq27	chr4:181442365-181482588	31978	34.3%	9.54826	8.23067	-0.5822	+0.0973
harseq28	chr4:62340702-62380806	31306	35.3%	8.07049	6.56390	-0.8043	+0.3014
harseq29	chr5:3811279-3851396	33416	43.4%	6.97228	5.00758	-1.1942	-0.7388
harseq30	chr1:61201972-61242077	32980	43.3%	10.45904	8.13716	-0.9192	-0.0731
harseq31	chr7:69265954-69306113	26656	37.5%	4.50345	3.45943	-0.9556	-0.5319
harseq32	chr16:72076474-72116585	33688	42.5%	10.74690	9.01213	-0.6689	+0.5535
harseq33	chr22:45599885-45640001	33130	47.7%	11.17433	10.69690	-0.1810	+0.5082
harseq34	chr9:13721078-13761218	33640	39.5%	12.44134	10.14241	-0.7714	-0.5499

region	hg18 coordinates	bpExp	AncGC	θ_W	θ_π	tajD	faywuH
harseq35	chr16:78693183-78733305	32027	40.1%	16.58456	14.88964	-0.4272	+0.1637
harseq36	chr2:118506031-118546191	33998	40.1%	6.27730	4.19485	-1.3515	+0.5153
harseq37	chr2:157528230-157568403	25637	35.2%	4.40280	4.22340	-0.1696	+0.6747
harseq38	chr3:4729793-4769893	36699	44.1%	8.51834	6.69460	-0.8880	-0.3448
harseq39	chr4:180887168-180927388	31479	34.4%	9.19176	8.60640	-0.2738	-0.1130
harseq40	chr11:113584984-113625403	37162	48.0%	9.84877	9.28639	-0.2400	+0.4624
harseq41	chr5:164645145-164685339	30097	35.1%	5.61116	5.06533	-0.4159	+0.1547
harseq42	chr18:74271167-74311322	31049	44.1%	13.08162	10.95373	-0.7070	-0.8094
harseq43	chr1:49106429-49146547	32260	41.2%	8.80935	7.33245	-0.6991	-0.6608
harseq44	chr3:60287955-60328104	34600	37.7%	12.39063	9.64907	-0.9265	-1.1410
harseq45	chrX:24838813-24878974	32865	37.3%	4.13205	3.00131	-1.1776	+0.9046
harseq46	chr6:50289961-50330074	28916	37.1%	8.70718	6.85926	-0.8960	-0.3163
harseq47	chr18:28585291-28625401	34980	38.7%	4.17305	3.28387	-0.8823	+0.1675
harseq48	chr11:79379569-79419751	32830	38.1%	12.89339	10.92111	-0.6376	-0.2361
harseq49	chr7:8892953-8933099	33979	34.6%	14.04067	12.25491	-0.5426	+0.2875
ctlreg50	chr6:92573969-92614071	33130	35.0%	7.08374	7.92122	+0.4997	+0.4966
ctlreg51	chr18:29440744-29481049	35292	34.8%	6.85577	5.05185	-1.0960	+0.4690
ctlreg52	chr13:94136843-94177447	30452	44.2%	8.08311	6.19251	-0.9619	-0.2070
ctlreg53	chrX:25290711-25330936	29942	38.0%	0.92511	0.47800	-1.8153	+0.0484
ctlreg54	chr1:67144380-67184529	26824	37.1%	4.11128	3.28860	-0.8321	+0.2590
ctlreg55	chr22:29998436-30038555	26253	44.8%	5.55553	4.19161	-1.0319	-0.3067
ctlreg56	chr2:162860968-162901074	30737	37.8%	4.69793	5.13615	+0.3892	-0.3816
ctlreg57	chr6:94005763-94046078	35030	32.6%	5.91775	5.47947	-0.3254	-0.7443
ctlreg58	chr7:77793163-77833324	33542	38.5%	10.12602	11.07011	+0.3987	+0.3906
ctlreg59	chr10:120111544-120151708	34946	39.2%	7.37268	5.95936	-0.7899	-0.3076
ctlreg60	chr17:69855000-69895000	26845	55.4%	6.66683	6.15304	-0.3400	+0.1802
ctlreg61	chr4:1080737-1121092	30674	43.1%	9.44086	10.76353	+0.6118	+0.1627
ctlreg62	chr7:86780083-86819921	26673	39.4%	8.53916	8.06259	-0.2416	-0.9293

Supplementary Table 2. Experiment Region Test Results segsites (tot): total number of segregating sites from our samples. fixed diffs (tot): number of positions for which we had sufficient coverage to make a genotype call in at least one sample that have a fixed difference between the human and chimp reference, excluding segregating sites and sites in dbSNP129. w2s,s2w: subsets with weak-to-strong or strong-to-weak mutations from the ancestral basepair. MWUpvalue,MWUoffset: p-value and estimated location offset (normalized to 22 samples) from Mann-Whitney U test comparing the frequency spectra of segsites w2s vs s2w, with positive offset values indicating W2S mutations shifted to higher derived allele frequencies. MKpval: p-value from McDonald-Kreitman-like test comparing the counts in w2s,s2w categories of segregating and fixed sites. SweepFinder: p-values for maximum CLR from SweepFinder run on the region with a background SFS derived from segregating sites pooled from all regions (Hpvalue) or from the same population segregating in Seattle SNPs resequenced regions (Spvalue).

region	segsites			fixed diffs			MWU		MK	SweepFinder	
	tot	w2s	s2w	tot	w2s	s2w	pvalue	offset	pvalue	Hpvalue	Spvalue
harseq1	105	19	71	113	49	54	0.03562	+1.25100	0.00015	0.208333	0.268519
harseq2	81	28	45	264	117	119	0.87810	-0.00088	0.10775	0.178664	0.252926
harseq3	107	34	64	504	194	235	0.34200	+0.30440	0.07031	0.130911	0.157123
harseq4	79	32	43	264	116	116	0.10850	+0.99850	0.28968	0.213721	0.196742
harseq5	74	30	30	286	170	80	0.60410	+0.15390	0.01073	0.146515	0.171717
harseq6	227	75	97	433	163	180	0.19460	+0.24840	0.45355	0.342159	0.293140
harseq7	55	16	26	356	158	153	0.28720	+1.05100	0.14016	0.539808	0.389727
harseq8	87	32	42	429	185	191	0.28970	+0.38070	0.37476	0.169000	0.121795
harseq9	99	33	50	278	132	109	0.41410	+0.00081	0.02186	0.049294	0.047896
harseq10	103	37	44	284	142	109	0.66870	-0.15340	0.09644	0.091280	0.130811
harseq11	62	26	27	268	172	60	0.16190	+0.90970	0.00079	0.049800	0.043100
harseq12	110	47	51	256	125	91	0.76910	-0.00218	0.11252	0.132699	0.098460
harseq13	94	47	35	249	100	100	0.77740	-0.00034	0.29474	0.193300	0.213457
harseq14	170	69	76	364	169	146	0.14120	+0.57070	0.23026	0.314890	0.383061
harseq15	94	43	39	308	158	116	0.48850	+0.48120	0.44683	0.204500	0.245441
harseq16	60	31	20	294	139	114	0.15180	+0.62490	0.53670	0.052200	0.055000
harseq17	67	25	28	200	87	84	0.36710	-0.09821	0.75341	0.209242	0.276955
harseq18	89	46	30	286	143	101	0.02016	+2.99900	0.79085	0.142700	0.155217
harseq19	76	28	35	321	177	96	0.78680	-0.00083	0.00394	0.256096	0.321658
harseq20	119	57	47	356	159	143	0.01450	+1.09100	0.73341	0.359765	0.347777
harseq21	103	54	41	330	170	109	0.00090	+2.00000	0.54473	0.365984	0.323354
harseq22	110	45	52	379	187	133	0.12150	+0.74970	0.04702	0.162441	0.170950
harseq23	94	47	30	361	175	131	0.18920	+0.44300	0.60600	0.324100	0.256926
harseq24	66	28	28	178	95	56	0.94760	-0.00109	0.11148	0.113100	0.034600
harseq25	124	53	50	370	196	123	0.00627	+1.65000	0.08404	0.025508	0.036177
harseq26	82	41	30	274	119	115	0.11550	-2.07600	0.34352	0.115100	0.144181
harseq27	105	54	35	383	195	125	0.01303	+1.15600	1.00000	0.096467	0.123767
harseq28	84	34	37	355	159	144	0.54860	+0.12300	0.51153	0.440232	0.412512
harseq29	79	26	45	529	224	223	0.62330	+0.10110	0.04060	0.100180	0.199323
harseq30	122	43	58	343	136	151	0.15780	-0.22180	0.41890	0.463826	0.435339
harseq31	41	19	17	181	79	75	0.53440	+0.10140	1.00000	0.140842	0.213764
harseq32	128	58	51	339	152	133	0.02369	+0.22340	1.00000	0.263714	0.313676

region	segsites			fixed diffs			MWU		MK	SweepFinder	
	tot	w2s	s2w	tot	w2s	s2w	pvalue	offset	pvalue	Hpvalue	Spvalue
harseq33	127	45	69	484	183	234	0.72950	+0.00052	0.45504	0.353104	0.445214
harseq34	147	63	47	432	229	141	0.00014	+1.64900	0.43624	0.152961	0.144700
harseq35	187	69	76	455	190	185	0.04483	+0.99920	0.55804	0.517048	0.358278
harseq36	76	27	34	359	192	127	0.62720	+0.00050	0.02390	0.286800	0.435435
harseq37	38	22	14	189	96	69	0.34520	+0.84850	0.85240	0.657600	0.506900
harseq38	110	42	49	478	273	140	0.94570	-0.00082	0.00050	0.189211	0.266641
harseq39	97	58	26	439	201	169	0.63830	-0.20110	0.01470	0.175035	0.293073
harseq40	127	47	61	285	108	140	0.53890	+0.16800	1.00000	0.408640	0.478168
harseq41	56	35	14	340	167	102	0.12600	+1.10000	0.25923	0.077339	0.116258
harseq42	135	47	72	522	247	205	0.07067	+1.07000	0.00383	0.154276	0.203180
harseq43	99	42	41	235	108	96	0.01237	+0.99890	0.79455	0.069247	0.078220
harseq44	150	78	44	447	204	174	0.24750	+0.22290	0.05902	0.112089	0.090924
harseq45	44	23	16	169	84	64	0.62450	-0.06291	0.85704	0.663900	0.788800
harseq46	86	32	40	380	149	180	0.02302	+1.10100	1.00000	0.131113	0.179274
harseq47	50	23	21	264	122	99	0.12030	+0.60890	0.74257	0.175853	0.222767
harseq48	149	65	64	321	152	112	0.88340	+0.00131	0.19554	0.203306	0.119695
harseq49	163	80	52	496	235	149	0.06355	+0.57090	0.91787	0.337953	0.322297
ctlreg50	80	33	33	427	201	163	0.75310	+0.19770	0.50213	0.147700	0.230143
ctlreg51	84	39	27	300	135	130	0.47400	+0.09916	0.27114	0.169100	0.213920
ctlreg52	87	27	49	193	60	94	0.59530	+0.00164	0.66584	0.190000	0.275341
ctlreg53	9	4	4	237	91	117	NA	NA	0.73305	0.100712	0.179191
ctlreg54	37	16	15	219	89	94	0.25880	+0.86680	0.84675	0.168881	0.213164
ctlreg55	49	20	23	185	81	69	0.42020	-0.26050	0.39345	0.063600	0.099000
ctlreg56	49	16	25	299	124	127	0.78910	+0.29070	0.24097	0.120012	0.138914
ctlreg57	67	31	28	324	164	118	0.93340	+0.00034	0.47059	0.099700	0.118300
ctlreg58	115	49	45	262	134	92	0.71610	-0.12350	0.26503	0.361595	0.300041
ctlreg59	91	31	50	318	138	134	0.60050	+0.00129	0.05735	0.103200	0.130054
ctlreg60	57	15	34	239	92	119	0.06740	+1.97400	0.10858	0.350521	0.336259
ctlreg61	95	35	48	309	147	124	0.80650	+0.00011	0.06024	0.111247	0.158769
ctlreg62	75	36	30	189	86	73	0.94860	+0.00088	1.00000	0.275100	0.242600

Supplementary Table 3. Mapping Statistics M: million reads. nTot: the total number of reads obtained for a given sample. nMap: the subset of nTot that mapped successfully to the human genome. mapFrac is nMap/nTot. nProbe: the subset of nMap that mapped to the regions defined by the probes on the Nimblegen enrichment array. probeFrac is nProbe/nMap. n4Best: the subset of nProbe that remain after filtering to allow at most 4 reads from each strand at each starting position in the genome. 4bestFrac is n4Best/nProbe. Sample DY02 was not included in the analysis because 97% of its on-target reads were removed in the "4best" pileup elimination step.

sample	nTot	nMap	mapFrac	nProbe	probeFrac	n4Best	4bestFrac
DY01	37.7M	18.7M	49.6%	8.4M	44.8%	4.7M	56.0%
DY02	37.4M	20.8M	55.6%	10.0M	48.3%	0.3M	3.3%
DY03	20.3M	12.1M	59.8%	5.0M	41.2%	2.6M	51.3%
DY04	22.8M	13.5M	59.2%	6.3M	46.4%	2.8M	44.9%
DY17	30.8M	12.3M	40.1%	4.6M	37.0%	3.9M	86.1%
DY18	28.3M	12.4M	43.9%	4.5M	36.0%	3.8M	85.0%
DY19	24.7M	11.5M	46.7%	4.5M	39.3%	4.0M	89.1%
DY20	22.1M	10.8M	48.8%	4.6M	42.5%	4.0M	87.8%
DY21	47.5M	18.5M	39.0%	5.5M	29.6%	4.5M	82.2%
DY22	38.7M	15.9M	41.2%	4.7M	29.8%	4.0M	84.6%
DY23	24.8M	10.3M	41.6%	3.4M	33.4%	2.8M	80.5%
DY24	18.2M	7.8M	42.9%	2.6M	32.7%	2.0M	77.1%

Supplementary Table 4. Seattle SNPs Gene Region Test Results basepairs(Gene): total length of genic region. basepairs(Map): sum of basepairs in regions that were mapped for variation within genic region. AncGC: the GC percentage of the *ancestral* bases in Map basepairs. segsites (tot): total number of segregating sites in Map basepairs for our samples. fixed diffs (tot): number of positions in Map basepairs that have a fixed difference between the human and chimp reference, excluding segregating sites and sites in dbSNP129. w2s,s2w: subsets with weak-to-strong or strong-to-weak mutations from the ancestral basepair. Other annotations as in Table 2.

gene	basepairs		Anc	segsites			fixed diffs			MWU		MK
	Gene	Map	GC	tot	w2s	s2w	tot	w2s	s2w	pvalue	offset	pvalue
a4galt	32545	15279	55.4%	86	24	47	138	41	73	0.61870	-0.00066	0.87432
abca3	66986	35333	56.9%	64	16	38	441	124	257	0.03467	+4.42800	0.75648
abce1	34706	33216	35.6%	58	28	20	200	93	71	0.13010	-0.10090	0.86981
abcg2	72304	33680	39.7%	73	33	33	398	154	196	0.25930	-0.99980	0.41881
adamts13	40953	30895	57.0%	107	29	60	304	71	188	0.10370	+0.00066	0.41532
agrp	50298	22054	54.2%	46	8	32	143	38	91	0.13350	+2.07600	0.31045
alb	21071	20586	35.0%	47	13	29	216	95	86	0.30830	-0.99960	0.01589
apobec3f	15687	11656	55.4%	65	19	31	211	62	118	0.10130	+0.99890	0.73809
aqp1	17617	16229	56.9%	49	14	25	185	55	100	0.41730	+0.22250	1.00000
avpr1b	11677	11498	51.7%	73	15	39	151	66	63	0.18210	+2.00000	0.00528
b3galt3	25347	24488	40.2%	89	37	40	188	80	77	0.25980	+0.09920	0.78093
bsg	14723	10804	62.8%	58	11	37	149	45	73	0.16460	+2.00100	0.07108
c5	101767	70230	37.7%	204	72	102	0	0	0	0.79650	-0.00012	1.00000
casr	106761	43238	43.4%	149	50	79	0	0	0	0.13670	+0.79840	1.00000
cbr3	15597	12107	48.8%	52	12	31	119	40	68	0.81720	+0.12290	0.34473
cd151	14528	12025	63.9%	76	18	41	79	16	43	0.90720	-0.00045	0.83914
ceacam8	18605	17580	45.1%	45	16	25	142	66	60	0.96790	-0.00087	0.15337
cfh	99171	47677	34.0%	173	82	68	464	193	181	0.28190	+0.22180	0.56198
coch	19799	16154	41.2%	44	17	15	90	37	38	0.16890	+1.94200	0.83333
cpsf4	22411	17245	54.4%	56	9	39	134	37	79	0.91280	-0.00075	0.12566
cyb5r4	102958	56189	36.2%	174	66	77	0	0	0	0.56890	+0.00118	1.00000
eln	44453	35947	56.0%	51	13	33	291	95	149	0.07420	+1.99900	0.18693
eng	43681	35650	54.9%	116	33	69	302	112	162	0.29150	+0.09903	0.15293
ermap	23195	20591	43.8%	65	26	26	189	77	84	0.62460	+0.00106	0.87344
fgl1	34970	30447	38.0%	265	103	108	440	188	165	0.64970	+0.00107	0.33835
fut2	27835	14690	52.2%	63	14	37	127	64	51	0.26600	+1.00100	0.00124
gas6	46842	33500	57.5%	173	48	104	398	147	221	0.12820	+0.99960	0.07424
gbgt1	14627	10753	56.1%	60	10	39	106	41	51	0.51740	-0.37470	0.00557
gpr154	195672	64253	39.9%	241	81	110	0	0	0	0.06121	+0.90070	1.00000
hnf4a	34014	27602	51.2%	100	27	59	311	106	170	0.47390	+0.22150	0.25190
hpgd	35444	31888	36.6%	122	60	43	316	157	107	0.29440	+0.50000	0.90599
il1f5	12425	10604	46.6%	48	15	26	77	28	41	1.00000	-0.00027	0.69235
il1f6	19137	15794	38.1%	44	18	21	109	59	39	0.93230	-0.00004	0.18142
il1f8	40032	28539	41.7%	85	31	40	93	48	28	0.29710	+0.09913	0.02100
il1rl2	56358	47182	42.0%	179	59	100	475	196	207	0.15970	+0.61050	0.01452

gene	basepairs		Anc	segsites			fixed diffs			MWU		MK
	Gene	Map	GC	tot	w2s	s2w	tot	w2s	s2w	pvalue	offset	pvalue
lep	19824	15916	49.3%	53	11	34	178	59	99	0.81870	-0.00085	0.11435
lipe	29453	24319	55.6%	67	17	39	186	58	102	0.86410	-0.00120	0.51473
mcp	46973	38855	38.0%	76	32	36	323	132	129	0.01202	+2.99900	0.68326
ncoa1	190138	150195	36.2%	285	132	112	0	0	0	0.47280	+0.00059	1.00000
nr1i2	39570	32317	46.8%	135	42	74	305	108	156	0.12080	+0.99950	0.42585
opn4	15754	15219	58.3%	81	20	56	115	32	68	0.88840	-0.00191	0.50505
otud4	44225	33213	37.8%	45	15	18	185	72	77	0.01354	-1.00000	0.84818
pan3	123999	44088	35.0%	105	50	45	0	0	0	0.06394	+0.89940	1.00000
pcyt1a	52884	24373	44.0%	48	10	29	169	84	66	0.55830	+0.29490	0.00104
pcyt1b	93091	51853	43.8%	82	32	38	354	125	191	0.62700	-0.00028	0.34980
pla2g7	34783	32965	40.4%	72	26	36	329	120	153	0.19290	+0.37380	0.88729
ppargc1a	102003	72261	37.9%	188	86	78	0	0	0	0.23470	+0.22160	1.00000
psd4	32965	31528	52.7%	145	39	82	389	173	161	0.90660	-0.00190	0.00028
ptgdr	12937	12659	43.2%	55	20	28	144	71	53	0.19210	+0.22160	0.08822
ptger2	18108	13546	42.5%	50	21	23	85	34	35	0.94370	+0.00111	1.00000
ptges	18536	15523	53.4%	53	17	25	179	67	91	0.21710	+0.83480	0.86195
rab38	65543	26305	39.2%	95	40	42	240	98	109	0.85610	+0.00106	0.89621
rap1b	53480	26072	39.0%	65	31	24	195	88	85	0.25240	+0.15490	0.53655
sema7a	27865	25552	57.5%	74	19	43	159	50	87	0.59900	+0.00148	0.52032
serpina10	13108	12602	46.1%	44	16	22	152	55	75	0.00588	+4.00100	1.00000
slc14a1	31111	27585	43.4%	146	54	69	266	99	131	0.96520	+0.00004	0.91048
slc20a1	21840	18951	43.3%	45	17	23	177	90	57	0.64950	+0.12300	0.04675
slc4a1	22398	17921	57.4%	48	7	39	167	36	108	0.53430	+0.44300	0.22469
tcf1	27688	25550	52.3%	68	20	42	221	90	108	0.58900	+0.00118	0.07754
tnni3k	312959	103913	35.9%	291	126	116	0	0	0	0.55040	+0.00175	1.00000
vldlr	36676	34462	42.0%	141	69	45	407	238	105	0.04855	+1.00200	0.08527
vwf	179597	45551	50.6%	197	49	129	0	0	0	0.62640	+0.00153	1.00000
